# The Notch signaling pathway: a potential target for cancer immunotherapy

**DOI:** 10.1186/s13045-023-01439-z

**Published:** 2023-05-02

**Authors:** Xinxin Li, Xianchun Yan, Yufeng Wang, Balveen Kaur, Hua Han, Jianhua Yu

**Affiliations:** 1 Xi’an Key Laboratory of Stem Cell and Regenerative Medicine, Institute of Medical Research, Northwestern Polytechnical University, Xi’an, 710072 Shaanxi People’s Republic of China; 2 State Key Laboratory of Cancer Biology, Department of Biochemistry and Molecular Biology, Fourth Military Medical University, Xi’an, 710032 Shaanxi People’s Republic of China; 3 Cancer Institute, The First Hospital of Jilin University, Changchun, 130021, People’s Republic of China; 4 Department of Neurosurgery, McGovern Medical School, University of Texas Health Science Center at Houston, Houston, TX 77225 USA; 5 Department of Hematology and Hematopoietic Cell Transplantation, City of Hope National Medical Center, 1500 East Duarte, Los Angeles, CA 91010 USA

**Keywords:** Immune cells, Cancer immunotherapy, Notch signaling, Tumor-associated macrophages, Chimeric antigen receptor (CAR), Immune checkpoint

## Abstract

Dysregulation of the Notch signaling pathway, which is highly conserved across species, can drive aberrant epigenetic modification, transcription, and translation. Defective gene regulation caused by dysregulated Notch signaling often affects networks controlling oncogenesis and tumor progression. Meanwhile, Notch signaling can modulate immune cells involved in anti- or pro-tumor responses and tumor immunogenicity. A comprehensive understanding of these processes can help with designing new drugs that target Notch signaling, thereby enhancing the effects of cancer immunotherapy. Here, we provide an up-to-date and comprehensive overview of how Notch signaling intrinsically regulates immune cells and how alterations in Notch signaling in tumor cells or stromal cells extrinsically regulate immune responses in the tumor microenvironment (TME). We also discuss the potential role of Notch signaling in tumor immunity mediated by gut microbiota. Finally, we propose strategies for targeting Notch signaling in cancer immunotherapy. These include oncolytic virotherapy combined with inhibition of Notch signaling, nanoparticles (NPs) loaded with Notch signaling regulators to specifically target tumor-associated macrophages (TAMs) to repolarize their functions and remodel the TME, combining specific and efficient inhibitors or activators of Notch signaling with immune checkpoint blockers (ICBs) for synergistic anti-tumor therapy, and implementing a customized and effective synNotch circuit system to enhance safety of chimeric antigen receptor (CAR) immune cells. Collectively, this review aims to summarize how Notch signaling intrinsically and extrinsically shapes immune responses to improve immunotherapy.

## Introduction

The Notch signaling pathway, which is highly conserved across species, is implicated in numerous aspects of cancer biology, including the cancer stem cell phenotype, tumor angiogenesis, metastasis, and tumor immune evasion [1–5]. After decades of study, scientists have revealed that Notch signaling plays an essential regulatory role in immune cells and the tumor microenvironment (TME) [5, 6]. Its double-edged roles in anti-tumor or pro-tumor immune regulations involve modulating tumor-associated macrophages (TAMs), myeloid-derived suppressor cells (MDSCs), dendritic cells (DCs), and other immune cells in the TME.

Previous studies have shown that Notch signaling regulates the activation, infiltration, and phenotypic switching of various immune cells (e.g., macrophages, T cells, among others). In addition, a synthetic Notch (synNotch) receptor can customize the anti-tumor response programs of T cells to kill tumor cells in a precise and localized manner. The synNotch system can deliver non-native therapeutic antibodies [e.g., programmed cell death protein 1 (PD-1) antibodies, cytotoxic T lymphocyte-associated antigen-4 (CTLA-4) antibodies] as well as derived customized cytokines [interleukin 2 (IL-2) and secreted interleukin 12 (IL-12)] [7]. Therefore, modulation of Notch signaling may be able to coordinate with immune responses to tumor cells. In this review, we provide an up-to-date overview of existing and emerging findings of Notch signaling in immune cells and of TME-related immune responses. We also discuss potential therapeutic strategies for reducing unwanted side effects of Notch signaling and examine how Notch signaling might be redirected to improve immunotherapy.

## Components, basic function and inhibition of Notch signaling

Notch signaling is highly conserved through evolution as a determinant of cell fate by mediating direct contact between adjacent cells [5, 6]. Regulation of Notch signaling participates in numerous aspects of tumor biology, including tumor angiogenesis, maintenance of tumor stem cells, and the responses of immune cells (e.g., DCs, T cells, and macrophages) [1–5, 8–10]. Notch signaling is also regulated by a variety of mechanisms, including post-transcriptional regulation, glycosylation, transcriptional repression/activation, epigenetic modifications, as well as other mechanisms. Additionally, its activity can be modulated by different signaling pathways (e.g., AKT, RUNX1, SIRT6, and DEC1) [5, 6]. The Notch signaling pathway in mammals has three major components: (i) Ligands for binding the extracellular segments of Notch receptors (Jagged1, Jagged2, Dll1, Dll3, and Dll4); (ii) Notch receptors (Notch1, Notch2, Notch3, and Notch4); (iii) RBP-J-dependent canonical downstream effectors (e.g., Hes family proteins) and RBP-J-independent non-canonical downstream effectors of Notch signaling (e.g., Iκκ, NF-κB, and PI3K/AKT) [11, 12].

**Fig. 1:**
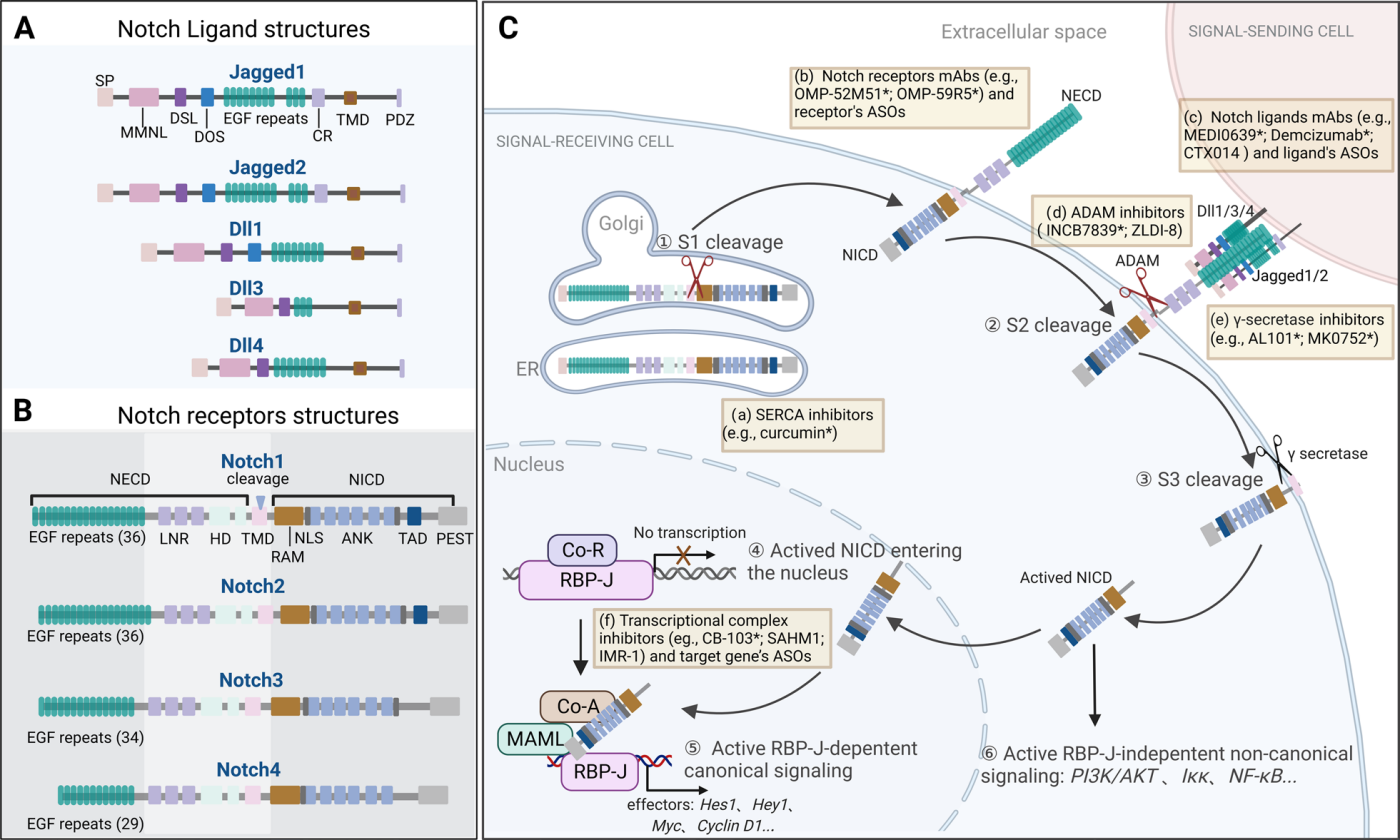
The Notch signaling pathway. **A-B** Composition of five Notch ligands (Jagged1, Jagged2, Dll1, Dll3, and Dll4) and four Notch receptors (Notch1, Notch2, Notch3, and Notch4). Each domain of Notch ligands and receptors is shown. The structures of Notch1-4 are highly homologous but show differences to certain extents. All of the four receptors have the same or similar LNR, HD, TMD, RAM, NLS, ANK, and PEST. The protein structures of Notch1 and Notch2 are highly similar, both of which have 36 EGF repeats in their NECDs. Compared with Notch1/2, Notch3 has 34 EGF repeats and lacks TAD structure, while Notch4 has only 29 EGF repeats and also lacks the TAD structure. SP: Signal peptide; MNNL: Module at N-terminal domain of Notch ligand; DSL: Delta, Serrate, and LAG-2 domain; DOS: Delta and OSM-11-like proteins domain; CR: Cysteine-rich domain; TMD: Transmembrane domain; PDZ: PDZ domain; LNR: Lin 12-Notch repeats; HD: Heterodimerization domain; RAM: RBP-J association module; NLS: Nuclear localization sequences; ANK: Ankyrin repeats; TAD: Transcription activation domain. PEST: proline, glutamic acid, serine, threonine-rich domain. **C.** Activation of Notch signaling: ①: Notch receptors are synthesized and processed in the ER and Golgi apparatus and then transported to the cell membrane to form heterodimers; ② and ③: Notch ligands from signal-sending cells bind to the NECD of signal-receiving cells. The binding triggers cleavage by ADAM and then γ-secretase, which releases activated NICD; ④ and ⑤: Activated NICD enters the nucleus and binds to MAML, RBP-J, and other proteins to form transcription complexes that promote the transcription of a series of genes (e.g., Hes1, Hey1) through RBP-J-dependent canonical Notch signaling; ⑥: Activated NICD directly activates the expression of a series of genes (e.g., PI3K/AKT) through RBP-J-independent non-canonical signaling. The six yellow rectangular boxes (a-f) represent clinically or preclinically used inhibitors, blocking antibodies, or target gene’s antisense oligonucleotides (ASOs) that inhibit Notch signaling. They are mainly: (a) inhibitors that inhibit the formation of Notch receptors (e.g., SERCA inhibitor); (b-c) antibodies that inhibit Notch receptors or ligands [e.g., OMP-52M51 (anti-Notch1), OMP-59R5 (anti-Notch2/3), MEDI0639 (anti-Dll4), Demcizumab (anti-Dll4), and CTX014 (anti-Jagged1/2)], and receptor’s or ligand’s ASOs; (d-e) ADAM inhibitors or γ-secretase inhibitors that inhibit the cleavage of Notch receptor (e.g., INCB7839, ZLDI-8, AL101, and MK0752); (f) Inhibitors that inhibit transcriptional complexes (e.g., CB-103, SAHM1, and IMR-1) and target gene’s ASOs. mAbs: monoclonal antibodies. Asterisks (*) indicate drugs that are being assessed in clinical trials. Figure was created with BioRender.com

The Notch signaling pathway is assembled and triggered via complex mechanisms. (i) The Notch receptor protein, a type I transmembrane protein originally synthesized in the endoplasmic reticulum (ER), is transported into the Golgi apparatus, cleaved into two fragments by furin, and then transported to the cell surface to form a heterodimer [6, 13]; (ii) Binding of ligands from signal-sending cells to the extracellular domain of Notch receptor (NECD) of signal-receiving cells, or activation of ligand-independent Notch receptors, causes the receptors’ extracellular subunits to dissociate from its transmembrane subunits, thereby releasing the activated intracellular domain of Notch receptors (NICD) [12, 14]; (iii) Activated NICD enters the nucleus and complexes with other proteins [e.g., recombination signal binding protein for immunoglobulin kappa (κ) J region (RBP-J) and mastermind-like (MAML)], to form a transcription complex, thereby regulating gene transcription (e.g., of Hes1 and Hey1 genes) [11, 13]; (iv) Also, activated NICD can directly activate the expression of genes (e.g., PI3K/AKT) through non-canonical regulations. The structures of Notch ligands and receptors, their basic functions of Notch signaling are summarized in Fig. 1A–C.

Previous studies have revealed that Notch signaling regulates the fate choice of various cells under physiological conditions [6], whereas dysregulated Notch signaling, especially abnormal activation, can promote the development of various malignancies. Therefore, in the past decades, drugs (mainly specific inhibitors or blocking antibodies) against Notch signaling are being tested in clinical trials or preclinical research for both solid and hematological malignancies [5, 12, 15–25]. The specific inhibitors and blocking antibodies of Notch signaling in clinical trials or preclinical studies are summarized below (Tables 1 and 2).

**Table 1:** Clinical trials desinged to target Notch signaling

Drug Name	Class	Identifier/Refs; Phase	Cancer	First posted; Status; Country	Results
Curcumin	SERCA inhibitor	NCT00094445 [26];II	Pancreatic cancer	2004; Completed; USA	25 patients took curcumin orally, 21 patients evaluable for response, and 2 patients had clinical biological activity. Among them, 1 patient remained stable condition > 18 months, another patient experienced transient but significant 73% tumor regression
		NCT01490996 [27, 28];I/II	CRC	2011; Completed; United Kingdom	Based on the FOLFOX chemotherapy measure, daily oral curcumin is safe and tolerable in CRC patients
		NCT02064673; III	PCa	2014; Recruiting; USA	Ongoing
OMP-52M51 (Brontictuzumab)	Anti-Notch1 mAb	NCT01703572 [29]; I	Lymphoid malignancies	2012; Completed; USA	In 24 assessable patients, OMP-52M51 treatment was generally well tolerated, and exhibited moderate anti-tumor activity with one PR and two SD. However, diarrhea was the main side effect of OMP-52M51
		NCT01778439 [30]; I	Solid tumor	2013; Completed; USA	Clinical benefit was seen in 6 of 36 (17%) assessable patients, 2 patients had PR and 4 patients had prolonged (≥ 6 months) SD. OMP-52M51 treatment was well tolerated in patients, and diarrhea was the main toxicity
		NCT02662608 [31]; I	ACC	2016; Completed; USA	Only 1 ACC patient with Notch1-mutant received OMP-52M51 treatment, and this patient had PR
		NCT03031691; I	Metastatic CRC	2017; Completed; USA	Unpublished
OMP-59R5	Anti-Notch2/3 mAb	NCT01277146 [32]; I	Solid tumor	2011; Completed; USA	Among 42 patients, three strategies of OMP-59R5 treatment (weekly dose < 2.5 mg/kg, every other or every third week dose 7.5 mg/kg) were well tolerated. The most common AE was GI toxicity, including diarrhea (81%), fatigue (48%), nausea (45%), anorexia (38%), vomiting (38%) and abdominal pain and constipation (24% each)
		NCT01647828 [33]; I/II	Stage IV pancreatic cancer	2012; Completed; USA	In metastatic PDAC, first-line drugs (e.g., nab-paclitaxel and gemcitabine) combined with OMP-59R5 did not improve OS, PFS, or ORR. PFS was specifically statistically worse in OMP-59R5-treated patients
		NCT01859741; I/II	Stage IV SCLC	2013; Terminated; USA	Terminated due to unimproved PFS
Rovalpituzumab tesirine (Rova-T)	Anti-Dll3 mAb	NCT01901653 [36]; I/II	Recurrent SCLC	2013; Completed; USA	82 patients received at least one dose of Rova-T. 11 of 60 (18%) assessable patients had a confirmed objective response, including 10 of 26 (38%) Dll3-high patients. Drug-related serious AEs occurred in 28 of 74 (38%) patients. Rova-T treatment showed encouraging single-agent anti-tumor activity with a manageable safety profile
		NCT02874664 [34]; I	SCLC	2016; Completed; USA	46 patients received at least one dose of Rova-T treatment. After administration of Rova-T, there were no clinically changes in QRS or PR intervals, electrocardiogram waveforms, or heart rate
		NCT02674568 [35]; II	SCLC	2016; Completed; USA, France	In 339 patients, ORR was 12.4%, 14.3%, and 13.2% in all, Dll3-high, and Dll3-positive patients, respectively. Median OS was 5.6 months in all patients, and 5.7 months in Dll3-high patients. The most common AEs were fatigue, photosensitivity reaction, and pleural effusion
Rovalpituzumab tesirine (Rova-T)	Anti-Dll3 mAb	NCT02709889 [40]; I/II	MCC	2016; Terminated; USA	In 65 patients, 1 MCC patient with Dll3‐high expression was treated with Rova-T and achieved partial positive response
		NCT02819999 [37]; I	SCLC	2016; Terminated; USA	Patients who received both Rova-T and platinum-based chemotherapy did not have better therapeutic benefits than patients who received platinum-based chemotherapy alone
		NCT03086239 [42]; I	SCLC	2017; Completed; Japan	In 29 Japanese patients, Rova-T treatment exhibited manageable toxicity. In Dll3-high expression patients, 3 of 18 (17%) patients had confirmed PR. The disease control rate was 56%, median PFS was 2.9 months, and median OS was 7.4 months
		NCT03026166 [38]; I/II	SCLC	2017; Terminated; USA	Rova-T in combination with other chemotherapy drugs were not well tolerable in SCLC patients
		NCT03061812 [39]; III	SCLC	2017; Completed; USA	Compared with topotecan treatment patients, patients who received Rova-T treatment exhibited an inferior OS, higher rates of serosal effusions, photosensitivity reaction, and peripheral edema
		NCT03543358; II	Cancer	2018; Completed; USA	Unpublished
SC-002	Anti-Dll3 mAb	NCT02500914 [41]; I	SCLC	2015; Terminated; USA	In 35 enrolled patients received ≥ 1 dose of SC-002 treatment, 23 patients experienced serious AEs, 5 patients achieved a PR, and no patients achieved a complete response
MEDI0639	Anti-Dll4 mAb	NCT01577745 [43]; I	Solid tumor	2012; Completed; USA	In 20 patients, 1 melanoma patient had PRs, and 7 patients had stable disease lasting ≥ 12 weeks. The most common TrAEs were increased aspartate aminotransferase, increased BNP, and fatigue. No treatment-related deaths occurred
Demcizumab	Anti-Dll4 mAb	NCT01189968 [44]; I	NSCLC	2010; Completed; Australia	46 treatment-naive NSCLC patients were enrolled. After treatment of demcizumab, 20 of 40 (50%) evaluable patients had objective tumor responses. The common AEs of patients were hypertension and raised brain natriuretic peptide
		NCT01189929; I	Pancreatic cancer	2010; Completed; Australia	Unpublished
		NCT01952249[45]; I	Primary peritoneal carcinoma	2013; Terminated; USA	In 19 patients who were enrolled, no DLT was observed. ORR was 21%. The most common TEAE were diarrhea (68%), fatigue (58%), peripheral edema (53%), and nausea (53%)
		NCT02259582; II	NSCLC	2014; Completed; USA	Unpublished
		NCT02289898; II	Pancreatic cancer	2014; Completed; USA	Unpublished
		NCT02722954 [46]; I	Advanced or metastatic solid tumor	2016; Completed; USA	In 27 patients, 1 patient was observed PR and 8 patients had stable disease. Demcizumab plus pembrolizumab were well tolerated in patients. However, there is no evidence to suggest that demcizumab has significant anti-tumor activity after treatment
INCB7839 (Aderbasib)	ADAM inhibitor	NCT04295759; I	High-grade gliomas	2020; Recruiting; USA	Ongoing
MK0752	γ-secretase inhibitor	NCT00100152; I	Leukemia	2004; Terminated; Unknown	Mediastinal masses decreased by 45% in 1/6 (16%) of patients; the study was discontinued due to severe diarrhea
MK0752	γ-secretase inhibitor	NCT00106145 [47]; I	Advanced BC or other solid tumor	2005; Completed; USA	103 patients received MK0752 treatment. Among patients with high-grade gliomas, 1 patient complete response and an additional 10 patients with stable disease > 4 months. The most common drug-related toxicities were diarrhea, nausea, vomiting, and fatigue
		NCT00645333 [48]; I/II	Metastatic BC	2008; Completed; USA	30 patients were treated with docetaxel plus escalating doses of MK0752. In tumors of patients undergoing serial biopsies, a decrease in BC stem cell markers (CD44^+^/CD24^−^, ALDH^+^) and mammosphere-forming efficiency was observed
		NCT01098344 [49]; I	Pancreatic cancer	2010; Completed; United Kingdom	44 eligible patients received MK0752 treatment with/without gemcitabine. Tumor response evaluation was available in 19 patients, 13 patients achieved stable disease, and 1 patient achieved a confirmed PR. MK0752 can combine with gemcitabine or as single-agent
LY900009	γ-secretase inhibitor	NCT01158404 [53]; I	Advanced cancer	2010; Completed; USA	In 35 patients who received LY900009, study drug-related AEs were diarrhea (46%), vomiting (34%), anorexia (31%), nausea (31%), and fatigue (23%)
PF-03084014	γ-secretase inhibitor	NCT02299635; II	TNBC	2014; Terminated; USA	SAEs 6/19; early termination of research due to project sponsors' reprioritization
AL101	γ-secretase inhibitor	NCT04461600; II	TNBC	2020; Active, not recruiting; USA	Ongoing
		NCT04973683; I	Adenoid cystic cancer	2021; Recruiting; USA	Ongoing
RO4929097	γ-secretase inhibitor	NCT01071564; I	BC	2010; Terminated; USA	Patients experienced life-threatening complications (e.g., arrhythmia) after treatment. Therefore, the clinical trial was terminated
		NCT01154452 [50]; I	Advanced or metastatic sarcoma	2010; Completed; USA	The combination of RO4929097 plus vismodegib was generally well tolerated. However, the combination did not meaningfully enhance the clinical efficacy
		NCT01196416; I/II	Recurrent or metastatic melanoma	2010; Completed; USA	Unpublished
		NCT01120275 [51]; II	Malignant melanoma	2010; Terminated; USA	In 32 evaluable patients, RO4929097 treatment was well tolerated. Specifically, 1 patient with confirmed PR lasting 7 months, another 8 patients with stable disease > 12 weeks, and 1 patient with stable disease > 31 months. The 6-month PFS rate was 9%, and the 1-year OS rate was 50%. The most common toxicities were nausea (53%), fatigue (41%), and anemia (22%)
		NCT01198184 [52]; I	Advanced solid tumor	2010; Completed; Canada	In order to evaluate the safety, PKs and pharmacodynamics of RO4929097 combined with temsirolimus, 17 patients were enrolled. 11 patients had stable disease. The most common toxicities included: fatigue (82%; grade 36%), mucositis, (71%;), neutropenia (59%), anemia (59%), and hypertriglyceridemia (59%)
		NCT01218620; I	Adult solid neoplasm	2010; Completed; USA	Unpublished
LY3039478	γ-secretase inhibitor	NCT02836600 [56]; I	Advanced solid tumor	2016; Active, not recruiting; Japan	In 11 enrolled Japanese patients, no dose-limiting toxicities or dose-limiting equivalent toxicities were observed. 1 patient (14.3%) with a desmoid tumor showed tumor size shrinkage of 22.4% and had stable disease for 22.5 months. The TrAEs are diarrhea, malaise, and vomiting
		NCT02784795 [54, 55]; I	Solid tumor	2016; Completed; USA, Spain Denmark, France,	LY3039478 combined with different anticancer agents (gemcitabine, cisplatin, and carboplatin) was poorly tolerated
CB-103	Notch transcription complex inhibitor	NCT03422679; I/II	Advanced solid tumors; hematological malignancies	2018; Terminated; USA	CB-103 was effective to control the Notch transcription complex, and is tolerable in patients with advanced tumors

CRC: Colorectal cancer; FOLFOX chemotherapy: folinic acid, fluorouracil and oxaliplatin combination chemotherapy for CRC; PCa:Prostate cancer; mAb: Monoclonal antibody; PR: partial response; SD: disease stabilization; ACC: Adenoid cystic carcinoma; AE: adverse event; GI: Gastrointestinal; PDAC: pancreatic ductal adenocarcinoma; OS: overall survival; PFS: progression-free survival; ORR: objective response rate; SCLC: Small cell lung cancer; MCC: Merkel cell carcinoma; TrAEs: treatment-related adverse events; BNP: brain natriuretic peptide; NSCLC: Non-small cell lung cancer; DLT: dose-limiting toxicities; TEAEs: treatment emergent adverse events; BC: Breast cancer; TNBC: Triple-negative breast cancer; SAEs: serious adverse events; PKs: pharmacokinetics

**Table 2:** Pre-clinical inhibitors or blocking antibodies that target Notch signaling

Name	Class	Type of tumor	Function	Reference
NVS-ZP7-4	Inhibitor that inhibits the synthesis of pre-Notch receptors	T-ALL	Interacts with ZIP7, increases ER Zn^2+^ levels, and inhibits the synthesis of pre-Notch receptors	[16]
FLI-06	Inhibitor that inhibits the synthesis of pre-Notch receptors	ESCC	Inhibits Notch trafficking and processing, and prevents the early secretion of Notch signaling	[23]
CTX014	Anti-Jagged 1/2 mAb	Solid tumor	Overcomes tumor-induced T cell tolerance, increases the infiltration of reactivated CD8^+^ T cells into tumors, and enhances the efficacy of T cell–based immunotherapy	[17]
ZLDI-8	ADAM inhibitor	HCC	Inhibits tumor growth in nude HCC-bearing mouse model	[18]
DAPT	γ-secretase inhibitor	HNSCC	Enhances tumor immunity in HNSCC	[19]
SAHM1	Notch transcription complex inhibitor	T-ALL	Suppress genome-wide suppression of Notch-activated genes in leukemic cells	[20]
IMR-1	Notch transcription complex inhibitor	EAC	Inhibits the growth of Notch-dependent EAC patient-derived xenograft tumors	[21]

pre-Notch receptors: precursors of Notch receptors; T-ALL: T cell acute lymphoblastic leukemia; ER: Endoplasmic reticulum; ESCC: Esophageal squamous cell carcinoma; mAb: Monoclonal antibody; HCC: Hepatocellular carcinoma; HNSCC: Head and neck squamous cell carcinoma; EAC: Esophageal adenocarcinoma

### Inhibitors that inhibit the synthesis of Notch receptors

The precursors of Notch receptors (pre-Notch receptors) are originally synthesized, their S1 portion is cleaved in the ER and Golgi apparatus, and then the cleaved Notch receptors are transported into the cell surface to further integrate with their ligands [6]. Previous studies showed that the inhibition of sarcoendoplasmic reticulum Ca^2+^-ATPase (SERCA) or zinc transporter impaired pre-Notch receptors synthesis, rendering them to be potential therapeutic targets [24, 25].

Curcumin, a natural phenolic compound that binds and inhibits SERCA, has been tested in pancreatic cancer [26], colorectal cancer (CRC) [27, 28], and prostate cancer (PCa) clinical trials. These results showed that oral administration of curcumin is generally safe and tolerated in CRC patients [27, 28], and in pancreatic cancer, two  out of 21 patients showed clinical biological activity and one of the two experienced transient but significant 73% tumor regression [26]. NVS-ZP7-4, an inhibitor that inhibits zinc transporter in ER, has been tested in T-ALL at the preclinical stage [16]. FLI-06, an inhibitor that inhibits the secretion of pre-Notch receptors before leaving ER, has been tested in esophageal squamous cell carcinoma (ESCC) at the preclinical stage [23]. In both in vitro and in vivo studies, these two inhibitors have shown inhibitory effects on the synthesis of pre-Notch receptors, which is worth future testing in the clinic. The results of each clinical trials and preclinical studies are shown in Tables 1 and 2.

### Blocking antibodies of Notch receptors and Notch ligands

After Notch ligands binds to NECD of Notch receptors, the extracellular subunits of Notch receptors are dissociated from their transmembrane subunits, resulting in NICD release and activation [6]. Some blocking antibodies that block the function of Notch receptors or ligands have been developed.

*Blocking antibodies of Notch receptors*: (i) OMP-52M51 (also called brontictuzumab), an anti-Notch1 monoclonal antibody (mAb), was tested in the clinic to treat lymphoid malignancies [29], solid tumor [30], adenoid cystic carcinoma (ACC) [31], and metastatic CRC. Overall, OMP-52M51 was well tolerated, exhibiting moderate anti-tumor activity with one partial response (PR) and two stable disease (SD) in twenty-four lymphoid malignancies [29], two PR and four SD in thirty-six (17%) assessable patients with a solid tumor [30], and one PR of one patient with Notch1-mutant ACC [31]. Diarrhea is the main toxicity after MP-52M51 treatment. (ii) OMP-59R5, an anti-Notch2/3 mAb, has been tested in clinical trials in solid tumor [32], stage IV pancreatic cancer [33], and stage IV small cell lung cancer (SCLC). Overall, the therapeutic effect of OMP-59R5 is not impressive. Either as a single agent or in combination with the first-line chemotherapy drugs (e.g., gemcitabine), OMP-59R5 did not improve overall survival (OS), progression-free survival (PFS), or objective response rate (ORR) of patients.

*Blocking antibodies of Notch ligands*: (i)–(ii) Rovalpituzumab tesirine (also called Rova-T) and SC-002, two anti-Dll3 mAbs, were each tested in multiple clinical trials, especially in SCLC (e.g., NCT01901653, phase I/II) [34–42]. Rova-T has controllable associated toxicities. In the treatment of SCLC, Rova-T exhibited moderate clinical activity. The results of clinical trial NCT01901653 showed that eleven of sixty (18%) evaluable patients received confirmed objective responses, including ten of twenty-six (38%) patients with high Dll3 expression [36]. Thus, it seems that Rova-T exhibits encouraging single dose anti-tumor activity with controllable safety, especially in patients with high Dll3 expression. (iii) MEDI0639, an anti-Dll4 mAb, was tested on clinical trials for solid tumors [43], and (iv) demcizumab, an anti-Dll4 mAb, was clinically tested in non-small cell lung cancer (NSCLC) [44], pancreatic cancer, primary peritoneal carcinoma [45], and other solid tumors [46]. For MEDI0639, in twenty solid tumor patients, only one melanoma patient with PRs; seven patients had stable disease lasting more than 12 weeks [43]; for demcizumab, in forty-six treatment-naive patients with NSCLC, twenty of forty (50%) evaluable patients had objective tumor responses [44]. CTX014, an anti-Jagged 1/2 mAb, was tested in solid tumor at the preclinical stage, and results showed that CTX014 treatment overcame tumor-induced T cell tolerance, increased the infiltration of reactivated CD8^+^ T cells into tumors, and enhanced the efficacy of T cell–based immunotherapy [17]. The results of each of clinical trials and pre-clinical studies are summarized in Tables 1 and 2.

### ADAM inhibitors and γ-secretase inhibitors

Two important cleaving enzymes, a disintegrin and metalloprotease (ADAM) and γ-secretase, catalyze the cleavage of Notch receptors [6]. Specifically, ADAM promotes the cleavage of NECD from the transmembrane (TM) NICD domain (S2 cleavage), while γ-secretase promotes the release of NICD from the TM domain (S3 cleavage), thereby achieving nuclear translocation [6, 12]. Therefore, ADAM and γ-secretase are important targets in blocking Notch signaling.

*ADAM inhibitors*: INCB7839 (also called Aderbasib), a small molecule drug targeting ADAM, has been proposed for a phase I clinical trial of high-grade gliomas (NCT04295759). This clinical trial is recruiting, and no results have been reported yet. The curative effect of other ADAM inhibitors (e.g., ZLDI-8) was tested in hepatocellular carcinoma (HCC)-bearing mice. Results showed that ZLDI-8 significantly inhibited tumor growth [18].

*γ-secretase inhibitors (GSIs)*: since 2004, at least six types of γ-secretase inhibitors have been clinically tested in various cancer patients. The details are as follows: (i) MK0752 was tested in clinical trials designed for leukemia, advanced breast cancer (BC) [47], metastatic BC [48], pancreatic cancer [49], or other solid tumors [47]. Overall, the main toxic side effect of this drug was diarrhea. But some patients also showed positive treatment reactions. In high-grade gliomas patients, one patient completely response and additional ten patients remained stable for more than four months to MK0752 treatment in one hundred and three patients in total [47]. Among forty-four eligible pancreatic cancer patients, thirteen patients achieved stable disease after MK0752 was combined with first-line chemotherapy drug gemcitabine, and one patient achieved a confirmed PR, indicating that MK0752 has the potential to be used in combination with first-line chemotherapy drugs [49]. (ii) RO4929097 has conducted clinical trials in BC, sarcoma [50], melanoma [51], adult solid neoplasm, and other solid tumors [52]. Overall, only one of thirty-two metastatic melanoma patients treated with RO4929097 achieved PR. Although RO4929097 is well tolerated, but it has significant toxicity. (iii)–(v) LY900009 in advanced cancer [53], PF-03084014 in triple-negative breast cancer (TNBC), and LY303947 in solid tumors [54–56] have also been tested. Overall, the clinical treatment effects of these three drugs were not impressive, and participants showed limited clinical responses. (vi) AL101 has been studied in clinical trials of TNBC and in adenoid cystic cancer. These two clinical trials are recruiting, and no results have been reported yet. DAPT has been tested in head and neck squamous cell carcinoma (HNSCC) at the pre-clinical stage. Results showed that DAPT decreased tumor burden in a mouse model after prophylactic treatment [19]. The results of each clinical trials and preclinical studies are shown in Tables 1 and 2.

### Notch transcription complex inhibitors

When activated NICD enters the nucleus, NICD binds with RBP-J and MAML to form a transcriptional complex, recruiting co-activators and triggering the transcription of Notch target genes [11, 13]. Therefore, targeted inhibition of the Notch transcription complex can also be an effective approach to block Notch signaling.

*Notch transcription complex inhibitors*: CB-103, the first drug to effectively control the Notch transcription complex, has been studied in advanced tumors and hematological malignancies (NCT03422679) in a phase I/II clinical trial. Results showed that CB-103 was well tolerated in cancer patients. The curative effect of the other two inhibitors, SAHM1 and IMR-1, has been tested in leukemic cells and an esophageal adenocarcinoma (EAC) patient-derived xenograft tumor model, respectively. Results showed that SAHM1 suppressed genome-wide suppression of Notch-activated genes in leukemic cells [20], and IMR-1 inhibited the growth of Notch-dependent EAC patient-derived xenograft tumors [21]. The results of each of clinical trials and pre-clinical studies are shown in Tables 1 and 2.

Collectively, among drugs that targeting Notch signaling, blocking antibodies of Notch ligands (e.g., anti-Dll3 mAb) and γ-secretase inhibitors (e.g., MK0752) have demonstrated encouraging therapeutic effects in clinical trials. Unfortunately, the therapeutic efficacy of other drugs does not seem to meet expectations, and further research is needed.

## Regulation of Notch signaling in immune cells

Numerous studies have confirmed that Notch signaling regulates cell development, cancer stem cell differentiation and proliferation, and cancer cell fate by targeting various genes [5, 57–60]. In the TME, the regulation of immune cell properties by Notch signaling also plays important roles in tumor progression, as reviewed below.

### Natural killer cells

**Fig. 2:**
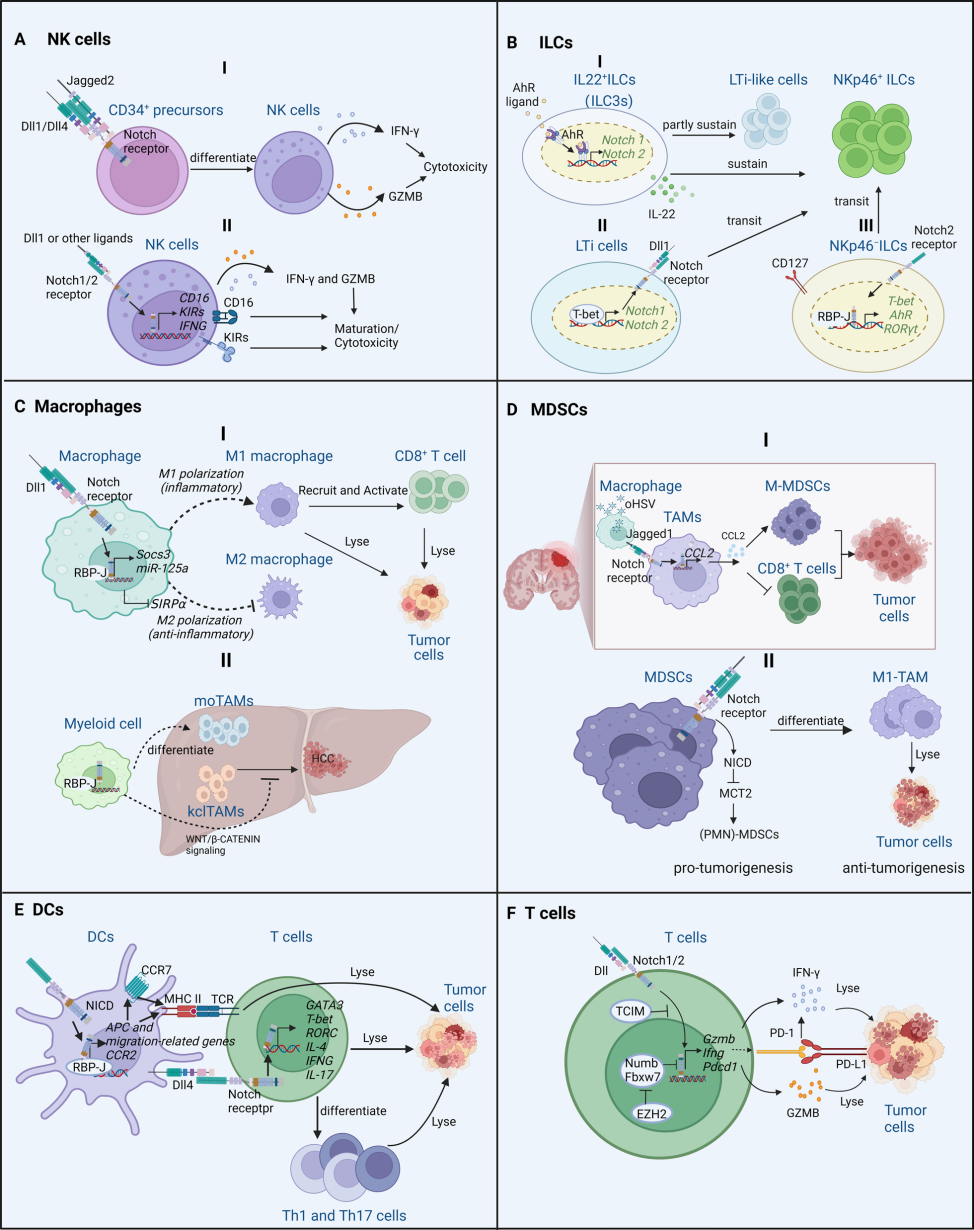
Mechanisms by which Notch signaling regulates anti- or pro-tumor functions of immune cells. **A. I:** After stimulation with Jagged2, Dll1, or Dll4, Notch signaling promotes the differentiation of CD34^+^ precursors into NK cells and enhances the anti-tumor properties of the NK cells by potentiating the secretion of IFN-γ and GZMB; **II:** Dll1 or other ligands that activate Notch1 or Notch2 increase the expression of *CD16*, *KIRs, IFNG*, enhance the maturation and cytotoxicity of NK cells. **B. I:** In IL22^+^ ILCs, AhR ligand enhances the transcription of *Notch1* and *Notch2* by activating AhR, sustains the NKp46^+^ ILC population, and, in part, also sustains the LTi-like cell population by activating Notch signaling; **II:** In LTi cells, T-bet promotes the transcription of *Notch1* and *Notch2* and also promotes the transition of LTi cells into NKp46^+^ ILCs; **III:** In NKp46^−^ ILCs, Notch activation mediated by Notch2 signaling promote the transcription of *T-bet*, *AhR,* and *RORγt* through canonical Notch signaling, which is mediated by RBP-J. This promotes the transition of NKp46^−^ ILCs into NKp46^+^ ILCs. **C. I:** Activation of canonical Notch signaling by Dll1 promotes the transcription of *Socs3* and *miR-125a* but inhibits the transcription of *SIRPα*, promoting M1 macrophage polarization and inhibiting M2 macrophage polarization; **II:** In the liver TME, myeloid cell-mediated canonical Notch signaling positively regulates the differentiation of moTAMs, but negatively regulates the proliferation of kclTAMs by regulating WNT − β-CATENIN signaling transmission in kclTAMs. **D. I:** In the glioma TME, oHSVs induce Jagged1 expression on macrophages; Jagged1-presenting macrophages spread activation of Notch signaling on TAMs, promotes the release of CCL2 from TAMs, recruits MDSCs, and then inhibits the cytotoxicity of CD8^+^ T cells; **II:** In MDSCs, activated NICD inhibits MCT2 expression, reduces the uptake of lactate from the TME, inhibits *Cox2* transcription, promotes MDSCs transition into M1(tumor-suppressive)-TAMs. **E.** Activation of RBP-J-mediated Notch signaling in DCs promotes the transcription of APC-related genes, migration-related genes, and CCR2; this activation enhances antigen presentation by DCs to T cells and accelerates tumor lysis. Dll4 of DCs activates Notch receptor of T cells, promotes the transcription of a series of genes (*GATA3*, *T-bet*, *RORC*, *IL-4*, *IFNG* and *IL-17*) that promote the differentiation of T cells or directly enhances the T cells’ cytotoxicity. **F.** Notch1/2 activated by Dll ligand promotes the transcription of *Gzmb*, *Ifng,* and *Pdcd1*, and enhances the anti-tumor ability of T cells by releasing IFN-γ and GZMB. However, Notch signaling activation  also inhibits the anti-tumor property of T cell*s* by increasing PD-1 expression. Transcriptional and immune response regulator (TCIM) inhibits Notch signaling, but enhancer of zeste homolog 2 (EZH2) activates Notch signaling by inhibiting the expression of Notch suppressors (Numb and Fbxw7). Figure was created with BioRender.com

Natural killer (NK) cells are crucial anti-viral and anti-tumor cells in the innate immune system [61, 62]. Early studies have found that Notch signaling plays an indispensable role in regulating their development and effector functions. For example, human umbilical cord blood (UCB) CD34^+^ precursors become committed to differentiate into NK cells in vitro after they are stimulated with Notch ligands (mainly Dll1, Dll4, and Jagged2) in the presence of cytokines [e.g., interleukin 7 (IL-7), Fms-like tyrosine kinase 3 (Flt3) ligand, and interleukin 15 (IL-15)]. These NK cells were able to lyse tumor cells because they upregulate their transcription and release of granzyme B (GZMB) and interferon-gamma (IFN-γ) [63–65]. As human NK cells mature, Dll1-mediated Notch signaling is activated to promote the expression of CD16 and killer Ig-like receptors (KIRs), resulting in cytotoxicity against tumor cells [66]. In human peripheral blood and decidual NK cells, activation of Notch1 and/or Notch2 by Dll1 and/or Dll4 promotes IFN-γ secretion [65]. Compared with normal human NK cells, Zakiryanova et al. found that the expression of Notch1 was significantly decreased in NK cells of patients with lung cancer or gastric cancer, while Notch2 was significantly reduced in patients with gastric cancer but not in those with lung cancer [67]. In murine NK cells, Kijima et al. found that DC-mediated NK cell activation was controlled by the interaction of Notch with Jagged2. Enforced expression of Jagged2 in DCs significantly enhanced the cytolytic effects of murine NK cells against YAC-1 cells by activating the NK cells’ Notch signaling [68]. Enforced expression of Jagged2 in A20 cells (a BALB/c-derived B cell lymphoma cell line with low expression of Jagged2) also enhanced the cytolytic efficacy of murine NK cells against A20 cells in vivo and in vitro [68]. Together, these observations suggest that Notch activation can significantly enhance the anti-tumor properties of NK cells. Therefore, targeted activation of Notch signaling in NK cells might be a promising strategy for enhancing NK cell therapy (Fig. 2A).

### Innate lymphoid cells

Innate lymphoid cells (ILCs) are newly discovered and defined lymphocytes involved in regulating innate and adaptive immune responses. They govern immune responses against viruses, intracellular pathogens, helminths, and tumors [69–71]. ILCs are widely distributed in various tissues and organs (e.g., liver, lymph nodes, small intestine lamina propria, and other mucosal tissues), and the various murine ILC lineages are distinguished by differences in transcription factor profiles and cytokine production. For example, T-bet [encoded by T-box transcription factor 21 (Tbx21)]-expressing group 1 ILCs (ILC1s) secrete mainly IFN-γ; GATA3-expressing group 2 ILCs (ILC2s) secrete mainly IL-5 and IL-13; and RORγt-expressing ILCs (ILC3s) secrete mainly IL-22 and IL-17. Murine ILC3s can be further divided into NKp46^+^ ILC3s, NKp46^−^ ILC3s, and lymphoid tissue-inducer cells (LTi cells) [72, 73].

During the past decade, researchers have uncovered multifaceted roles for Notch signaling in ILC subsets. In 2011, Possot et al. cultured murine bone marrow (BM) common lymphoid progenitors (CLPs) on OP9-Dll4 stroma (to activate Notch signaling) or in the presence of DAPT (to inhibit γ-secretase and therefore Notch signaling) and found that the maturation of adult BM-derived RORγt^+^ ILCs (also defined as ILC3s) was Notch2-dependent manner [74]. A subsequent study of murine gut ILC22 cells (now known as ILC3s), which include NKp46^+^ILCs [CD3^−^NKp46^+^NK1.1^lo–neg^RORγt^+^ cells (also defined as NKp46^+^ ILC3s)] and LTi cells, showed that Notch signaling was crucial for the downstream signaling of aryl hydrocarbon receptor (AhR) during the generation of murine NKp46^+^ ILCs (likely ILC3). In contrast, LTi-like cells were partly dependent on Notch signaling [75]. Compared with WT mice, RBP-Jκ-CD mice (conditional deletion of RBP-Jκ expression in the hematopoietic compartment) had considerably fewer NKp46^+^ ILCs in the lamina propria [75]. Mechanistically, the binding of AhR ligands on AhR promotes the translocation of AhR into the nucleus, where it binds to regulatory sites and promotes the expression of Notch receptors (mainly *Notch1*, *Notch2*), enhances IL-22 secretion, and ultimately sustains the NKp46^+^ ILC population and partly sustains LTi-like cells in small intestine (SI) lamina propria [75]. A subsequent study by Rankin et al. demonstrated that T-bet-mediated NKp46^+^ ILC development could also be achieved via Notch (mainly *Notch1, Notch2*) signaling [76]. Specifically, after being exposed to Dll1 for 9 days, murine SI lamina propria Rorc(γt)^+/GFP^ *Tbx*21^+/+^ LTi cells generated NKp46^+^ ILCs, whereas Rorc(γt)^+/GFP^*Tbx21*^−/−^ LTi cells did not generate that subset in vitro. This suggests that Notch signaling plays an integral role in the T-bet-mediated transition of LTi cells into NKp46^+^ ILCs [76]. In addition, in murine SI lamina propria, RBP-J-mediated Notch2 signaling contributed to the transition of NCR^−^ ILC3 precursors (NKp46^−^ ILC3s) into NCR^+^ ILC3s (NKp46^+^ ILC3s) in a cell-autonomous manner. Mechanistically, activation of RBP-J-mediated Notch2 signaling mainly stimulates the expression of genes encoding transcription factors, such as T-bet, AhR, and RORγt [77]. These murine studies support the notion that Notch signaling regulates ILC3 plasticity by controlling the fate of NKp46^+^ cells. In human ILCs, researchers have also found that, in combination with IL-7, Notch signaling induces the differentiation of hematopoietic progenitor cell subpopulation one [HPC-1, CD45RA (RA)^−^Flt-3^+^c-Kit^hi^ cells] into NKp44^+^ ILC3s [78]. Thus, Notch signaling plays an essential regulatory role in the development and phenotypic transition of ILC subsets, mainly ILC3s. However, our understanding of the role of Notch signaling in ILC-mediated immune responses in cancer is still preliminary, as even ILCs’ role in cancer is not yet very clear. We recently started to elucidate the role of ILCs in cancer [79, 80]. Therefore, further studies that explore the role and regulatory mechanism of Notch signaling in immune responses mediated by ILCs are warranted (Fig. 2B).

### Macrophages

Macrophages are specialized phagocytes in innate immunity. As one of the first responders to infection, they recognize and degrade tumor cells [81]. In the TME, macrophages are extremely plastic, and their interaction with tumor cells and/or the stromal microenvironment usually polarizes M1-like tumor-associated macrophages (M1-TAMs) into M2-like tumor-associated macrophages (M2-TAMs) [82]. Generally, M1-TAMs promote anti-tumor inflammatory responses and exert anti-tumor effects [82, 83]. In contrast, M2-TAMs are involved in neovascularization [84] and matrix deposition and remodeling [85], and they participate in immunosuppression, promoting tumor growth [84]. Therefore, promoting the polarization of macrophages into M1-TAMs or reversing M2-TAMs into M1-TAMs are key strategies for targeting macrophages in cancer immunotherapy [82, 86, 87].

In recent years, our research has suggested that Notch signaling plays a crucial regulatory role in switching macrophage phenotypes and thus in remodeling the TME [88–91]. Specifically, some Notch signaling molecules (e.g., *Notch1, Notch2, Hes1*) were expressed higher in M1-TAMs than in M2-TAMs in a B16F10 melanoma in vivo model. Forced activation of Notch signaling by co-culture with OP9-Dll4 cells promoted anti-tumor activity by polarizing macrophages into IL-12-producing M1-macrophages but not into M2-macrophages. Mechanistically, knockout of RBP-J-mediated Notch signaling inhibited M1 polarization by inhibiting LPS-induced suppressor of cytokine 3 (*Socs3*) expression [88]. Using NIC transgenic mice controlled by Lyz2-Cre (NIC^CA^), we subsequently showed that forced activation of Notch signaling in macrophages in vivo repressed tumor growth while diminishing TAM phenotypes. Mechanistically, miR-125a has been identified as a key downstream miRNA of RBP-J-mediated Notch signaling activation. Overexpression of miR-125a promoted M1 polarization and suppressed M2 polarization, boosting anti-tumor activity [89]. In addition, signal regulatory protein α (SIRPα), a key inhibitor of macrophages, was identified as the key downstream molecule of RBP-J-mediated Notch signaling. Notch activation repressed SIRPα expression through the Hes family co-repressors and then enhanced tumor cell lysis partly by promoting polarization into the M1 phenotype. Soluble mSIRPα^ext^ polypeptides, which possess the extracellular domains of mouse SIRPα, promoted M1 polarization and increased phagocytosis of tumor cells by macrophages [90]. This study indicates that specifically activating Notch signaling to inhibit the SIRPα-CD47 axis might be a promising strategy for releasing macrophages from phagocytic inhibition. We recently generated a type 1 herpes simplex virus-based oncolytic virus (oHSV) that expresses a full-length anti-CD47 antibody (αCD47) to block the CD47 ‘don’t eat me’ signal. This engineered virus suppressed tumor growth in both glioblastoma and metastatic ovarian cancer models, partly by promoting the M1 polarization of macrophages [92, 93]. In the TME of murine orthotopic HCC, myeloid-specific RBP-J knockout significantly promoted the growth of orthotopic tumors [91]. Compared with control mice, the infiltration of CCR2-independent TAMs—mainly Kupffer cell-like TAMs (kclTAMs) but not monocyte-derived TAMs (moTAMs)—in the liver was significantly higher in RBP-J knockout mice [91]. Mechanistically, RBP-J deficiency in myeloid cells impeded the differentiation of moTAMs, but promoted the proliferation and pro-tumor cytokine [e.g., interleukin 10 (IL-10)] production of kclTAMs by upregulating WNT-β-CATENIN signaling, and then accelerating the progression of murine orthotopic HCC [91]. Together, these findings suggest that intrinsic activation of Notch signaling promotes the M1 polarization and suppressed M2 polarization of macrophage to boost anti-tumor activity, while intrinsic inhibition of Notch signaling promotes the proliferation of kclTAMs to boost pro-tumor activity (Fig. 2C).

### Myeloid-derived suppressor cells

Myeloid-derived suppressor cells (MDSCs) are another major immune response modifier in cancer, as they interfere with immune responses against tumors and facilitate tumor metastasis and angiogenesis [94]. According to the differences in cell surface markers, MDSCs can be divided into different subtypes. Granulocytic-MDSCs [G-MDSCs or polymorphonuclear (PMN)-MDSCs] and mononuclear MDSCs (M-MDSCs) are two important immunosuppressive subsets [95, 96].

Recently, Wang et al. showed that Notch signaling was significantly inhibited in PMN-MDSCs of tumor-bearing mice [97]. Compared with MDSCs of control mice, the MDSCs (mainly PMN-MDSCs) of mice with specific knockout of RBP-J in myeloid cells were significantly less immunosuppressive. Mechanistically, knockout of RBP-J inhibits the signal transducer and activator of transcription 3 (STAT3) signaling and reduces the inhibition capability of PMN-MDSCs on the proliferation and activation of allogenic T cells, while the deficiency of the Notch signaling has not much effect on M-MDSC [97]. Therefore, blocking RBP-J-mediated canonical Notch signaling, specifically in PMN-MDSCs, might be an ideal strategy for inhibiting tumor progression [97]. Using Cybersort and Gene Set Enrichment Analysis (GSEA), Otani et al. analyzed TCGA database and revealed that a higher Notch score positively correlated with M-MDSC recruitment in glioma patients [98]. Mechanistically, treating mice bearing intracranial glioma with oHSV induced Jagged1 expression on macrophages. These Jagged1-presenting macrophages spread Notch activation in the TME, especially in TAMs. TAMs with Notch activation induce the secretion of CCL2, further amplifying M-MDSCs recruitment and attenuating anti-tumor immune response of T cells [98]. Blockading Notch signaling with GSI (γ-secretase inhibitor) significantly reduced the M-MDSC-mediated immunosuppressive TME and activated CD8^+^ T cell-dependent anti-tumor memory response [98]. A study from our group showed that activating Notch signaling in murine myeloid cells significantly inhibited tumor progression. Activated NICD inhibited lactate import 2 (MCT2) expression via Hes1, thus reducing lactate intake in myeloid cells. Activated NICD also promoted the differentiation of M-MDSCs into M1-type TAM but not into PMN-MDSCs in the TME [99].

Together, these studies highlight the complex roles of Notch signaling in the differentiation of different MDSC subtypes, suggesting that targeting activation or inhibition of Notch signaling in MDSCs for cancer treatment must be context-dependent. However, oncolytic virotherapy combined with Notch blockade may be a promising strategy for synergistically inhibiting the immunosuppressive function of M-MDSCs and thereby enhancing therapeutic benefits in glioma or other tumors that respond positively to inhibition of Notch signaling (Fig. 2D).

### Dendritic cells

Dendritic cells are professional antigen-presenting cells (APCs) that can efficiently intake and process antigens, and then present them to T cells, leading to activation of adaptive immune responses against pathogens and tumors [100]. Meng et al. identified a new human DC subset that highly expresses the Notch ligand Dll4 (Dll4^+^ DCs) [101]. Compared with Dll4^−^ DCs, these Dll4^+^ DCs can better promote the differentiation and expansion of T helper (Th) cells (e.g., Th1 and Th17 cells) and effector CD8^+^ T cells because they upregulate the transcription of differentiation-related transcription factors (e.g., GATA3, T-bet, RORC) and the production of anti-tumor effector cytokines (e.g., IL-4, IFN-γ, and IL-17). This suggests that high levels of Dll4 in DCs indicate the high anti-tumor potential because of upregulated antigen presentation and adaptive immune responses [101, 102].

In addition to Notch ligands, we found that Notch receptors and their downstream effectors are essential for DCs’ effector function. Thus, activation of RBP-J-mediated Notch signaling was critical in DC-dependent anti-tumor immune responses [9]. Compared with murine RBP-J^+/−^ DCs, RBP-J^−/−^ DCs (specific knockout of RBP-J in DCs) lost inhibition of tumors (e.g., B16F10 melanoma, H22 hepatoma, and Lewis lung carcinoma) in vivo because DC migration and antigen presentation to T cells were inhibited [9]. During the progression of colitis-associated CRC, mice whose DCs were deficient in Notch signaling were more susceptible to the disease than mice with normal DCs [103]. In contrast, adoptive transfer of Notch-primed DCs in mice restrained the progression of inflammation-associated CRC [103]. Mechanistically, chemokine receptors [mainly CC-chemokine receptor 7 (CCR7)] of DCs were identified as a critical downstream component of RBP-J-mediated Notch2 signaling, and upregulation of CCR7 mediated by activated Notch2 signaling facilitated DC migration and cross-presentation of antigens to CD8^+^ T cells [103]. Kirkling et al. found that Notch signaling facilitated the differentiation and CCR7-dependent migration of conventional DCs (cDCs) and then promoted their cross-presentation of antigens to T cells [104]. In addition, Notch signaling can be activated in DCs by a polysaccharide [Lycium barbarum polysaccharide (LBP)] and can then induce the phenotypic and functional maturation of DCs to promote DC-mediated cytotoxicity of T lymphocytes (CTLs) [105]. In general, Notch signaling is a positive regulator of DC maturation, antigen presentation, and adaptive immune responses, but the specific regulation mechanisms remain to be explored (Fig. 2E).

### T cells

T cells, especially CD8^+^ T cells, are well known for their cytolytic effects that require prior sensitization during adaptive immune responses [106]. Previous reports indicated that activation of Dll1-mediated Notch signaling (mainly Notch2 signaling) promoted the differentiation and cytolytic function of murine T cells both in vitro and in vivo [107]*.* By using a Notch2^f/f^E8I-Cre^+^ mouse model (lacking Notch2 expression in peripheral CD8^+^ T cells but not in CD4^+^ T cells), the authors found that the knocking out of Notch2 inhibited (compared to the control) the differentiation of naive CD8^+^ T cells into CTLs and could not control the growth of OVA-expressing EG7 thymoma cells and EG7 cells in vivo. These observations indicate that Notch2 is crucial for the anti-tumor response of CTL cells [107, 108]. Using a ChIP assay to explore the mechanism, the authors found that a complex of activated NICD, phosphorylated-CREB1, and transcriptional coactivator p300 bound to the promoter of the *Gzmb* gene, enhancing its transcription [107, 108]. Of note, in vitro and in vivo tumor models (e.g., breast adenocarcinoma, lung cancer, thymoma) and three follow-up studies also demonstrated that activation of Notch signaling significantly enhanced the anti-tumor and/or anti-tumor memory capacity of CD8^+^ T cells by promoting the expression of IFN-γ and GZMB. In these studies, Notch signaling was activated by (i) using mice whose CD8^+^ T cells contained a specifically activated NIC (Notch1 intracellular domain) [109], (ii) treating CD8^+^ T cells with the proteasome inhibitor bortezomib [110], or (iii) treating CD8^+^ T cells with the Notch ligand Dll1 [111]. However, in a different TME (e.g., in murine HCC or ovarian cancer), Notch signaling was regulated by a series of genes [e.g., transcriptional and immune response regulator (1810011O10 Rik, also known as TCIM) and enhancer of zeste homolog 2 (EZH2)] to modulate the anti-tumor immune response of T cells. Specifically, high expression of TCIM in T cells inhibited the nuclear translocation of activated NICD of the Notch2 receptor and thus suppressed the activation of downstream effector molecules, thereby reducing the cytotoxicity of CD8^+^ T cells [112]. Restricting glucose uptake of T cells from TME inhibited EZH2 expression, which indirectly inhibited the Notch signaling through suppressing the two Notch repressors, Numb and Fbxw7, leading to dampening anti-tumor activity of T cells [113]. Together, these studies indicate that Notch signaling plays a positive regulatory role in the anti-tumor properties of T cells. Therefore, activating Notch signaling in T cells, especially CD8^+^ T cells, might be a good strategy for enhancing anti-tumor responses.

As well as boosting T cells’ anti-tumor effects, Notch signaling might accelerate T cell exhaustion. The transcriptional activation complex of canonical Notch signaling directly binds to the promoter of *Pdcd1* (encoding PD-1, a marker gene that promotes T cell exhaustion) to promote *Pdcd1* transcription in CD8^+^ T cells [114]. Compared with colorectal T cells from healthy individuals, the expression of PD-1 and Notch signaling molecules (*NOTCH1*,* NOTCH2*,* HES1*, and *HES*5) was elevated in tumor-infiltrating CD8^+^ T cells from CRC patients [115]. Inhibition of Notch signaling not only promoted the cytotoxicity of tumor-infiltrating CD8^+^ T cells, but also enhanced CD8^+^ T cells’ production of proinflammatory cytokines [including IFN-γ, tumor necrosis factor alpha-like (TNF-α), interleukin-1 beta (IL-1β), IL-6, and IL-8] in those patients. This process was accompanied by decreased PD-1 expression in CD8^+^ T cells but did not affect cell proliferation [115]. This result suggests that Notch signaling has potential immunosuppressive properties that might inhibit the cytolytic and non-cytolytic functions of CD8^+^ T cells by inducing PD-1 in colorectal cancer patients [115]. In addition, the single-cell RNA sequencing of T cells in the human TME (e.g., lung cancer, pan-cancer) demonstrated that RBP-J expression was also related to the cytotoxicity or exhaustion of T cells [116, 117]. Together, the above evidence suggests that Notch activation can significantly enhance anti-tumor properties but may also potentially promote T cell exhaustion. However, targeted activation of Notch signaling in T cells combined with immune checkpoint blockers (ICBs), such as αPD-1, might be a promising strategy for enhancing T cell therapy (Fig. 2F).

In summary, Notch signaling plays a “double-edged sword” role in regulating immune responses, as Notch signaling can modulate the functions of anti- or pro-tumor immune cells. Specifically, for innate immune cells (e.g., NK cells, DCs, and macrophages), activation of Notch signaling mainly: (1) enhances the anti-tumor property of NK cells directly; (2) promotes the maturation and antigen presentation of DCs; and (3) facilitates the transition of macrophages into an M1 type. All of these can inhibit tumor progression. However, in the different TME, Notch signaling plays different roles in MDSCs-mediated tumor immunity. In the TME of murine lung carcinoma model, activating Notch signaling in myeloid cells promotes the differentiation of M-MDSCs into M1-TAMs and thus inhibits tumor progression. However, in the glioma TME, activating Notch signaling promotes M-MDSC-mediated immunosuppression and thus facilitates tumor progression. For adaptive T immune cells (e.g., T cells), activation of Notch signaling enhances their anti-tumor property, but Notch signaling also potentially enhances the exhaustion of T cells by upregulating PD-1 expression.

## SynNotch can be used as a tool to increase T cell cytotoxicity and specificity

**Fig. 3:**
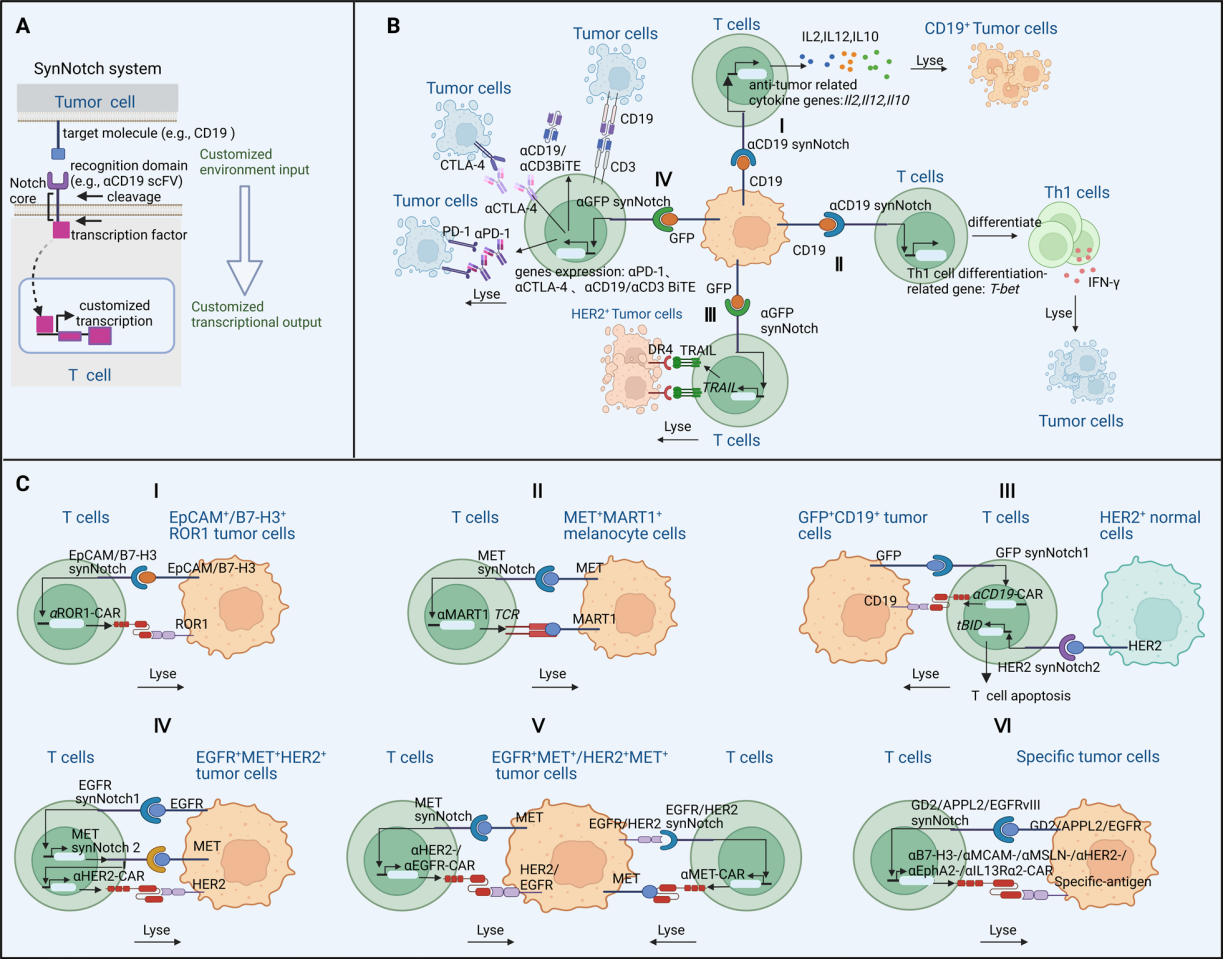
The synNotch system increases T cell specificity. **A**: Top, customized recognition domain of synNotch receptors (e.g., αCD19 scFV) detects a signal molecule (e.g., CD19) on target tumor cells. Middle, the core regulatory region of the Notch receptor that governs proteolysis or cleavage is activated by the interaction between the receptor and the tumor target signal (top), and a cytoplasmic orthogonal transcription factor is released; Bottom, the orthogonal transcription factor enters the nucleus of engineered T cells and controls the function-related transcriptional programs. **B. I:** αCD19-synNotch T cells recognize CD19^+^ tumor cells and release customized cytokines (e.g., IL-2, IL-12, and IL-10) that destroy CD19^+^ tumor cells;** II:** αCD19-synNotch T cells recognize CD19^+^ tumor cells, promote the transcription of differentiation-related genes (e.g., *T-bet*), and then promote T cells differentiation into Th1 cells; **III:** αGFP-synNotch T cells recognize GFP^+^ tumor cells, promote the transcription of *TRAIL*, and accelerate the lysis of tumor cells; **IV:** αGFP-synNotch T cells recognize GFP^+^ tumor cells, promote the production of antibodies (e.g., αPD-1, αCTLA-4 and αCD19/αCD3 BiTE), and accelerate tumor lysis. **C. I:** EpCAM/B7-H3-synNotch-αROR1-CAR-T cells recognize EpCAM^+^/B7-H3^+^ tumor cells, promote the expression of αROR1-CAR on T cells, and enable T cells to recognize and lyse EpCAM^+^/B7-H3^+^ROR1^+^ tumor cells; **II:** MET-synNotch-αMART1-T cells recognize MET^+^ tumor cells, and then enable T cells to lyse MET^+^MART1^+^ melanocytes; **III:** GFP-synNotch1-HER2-synNotch2-αCD19-CAR-T cells recognize GFP^+^ tumor cells, promote the expression of αCD19-CAR on T cells, and recognize and lyse GFP^+^CD19^+^ tumor cells. However, if GFP-synNotch1-HER2-synNotch2-αCD19-CAR-T cells recognize HER2^+^ normal cells, the proapoptotic factor tBID (truncated BH3-interacting domain death agonist) will be expressed by T cells to casue T cell apoptosis; **IV:** EGFR-synNotch1-MET-synNotch2-αHER2-CAR-T cells recognize two antigens from tumor cells (first one is EGFR and second one is MET). This promotes the expression of αHER2-CAR on T cells, and enable T cells to lyse EGFR^+^MET^+^HER2^+^ tumor cells; **V:** Both MET-synNotch-αHER2/αEGFR-CAR-T cells and EGFR/HER2-synNotch-αMET-CAR-T cells recognize and lyse EGFR^+^MET^+^/HER2^+^MET^+^ tumor cells; **VI:** GD2-synNotch-αB7-H3-CAR-T cells, APPL2-synNotch-αMCAM/αMSLN/αHER2-CAR-T cells, or EGFRvIII-synNotch-αEphA2/αIL13Rα2-CAR-T cells recognize the first antigen (GD2, APPL2, or EGFRvIII) on tumor cells, promote the expression of αB7-H3-CAR, αMCAM/αMSLN/αHER2-CAR, or αEphA2/αIL13Rα2-CAR on T cells, and enable T cells to lyse specific tumor cells (GD2^+^B7-H3^+^ tumor cells, APPL2^+^MCAM^+^/MSLN^+^/HER2^+^ tumor cells, or EGFRvIII^+^EphA2^+^/IL13Rα2^+^ tumor cells). Figure was created with BioRender.com

Adoptive T cell therapy, especially with CAR-T cells, has achieved unprecedented success against hematological malignancies [e.g., chronic lymphocytic leukemia (CLL), acute lymphoblastic leukemia (ALL), and lymphoma] but has shown only modest progress with solid tumors [118]. Historically, adoptive T cell therapy (especially with CAR-T cells) has been facing many challenges, inducing expected and unexpected toxicities (e.g., cytokine release syndrome, ‘on-target/off-tumor’ recognition), and is prone to exhaustion in the TME [119, 120]. Thus, many researchers are looking for strategies to overcome these obstacles [121]. In 2016, the Lim group took advantage of the Notch receptor’s unique structure to replace and customize its extracellular and intracellular domains, transcription factor domains, and downstream effectors. This led to the development of a synthetic Notch (synNotch) system, which allows engineered T cells to respond to tumor antigens in a very precise and localized way. The structure of synNotch system is shown in Fig. 3A [7]. The synNotch platform was used to engineer T cells that produce a customized therapeutic response after they encounter a tumor antigen. For example: (i) synNotch T cells can produce selective cytokines (e.g., IL-2 and IL-12) to precisely regulate immune responses; (ii) synNotch T cells can increase the expression of differentiation-related molecules (e.g., T-bet) to promote T cells differentiation into anti-tumor Th1 cells, thus controlling the fate choice of T cells; (iii) synNotch T cells can bind to specific receptors, trigger self-destruction of cancer cells and further promote their demise by producing TNF-related apoptosis-inducing ligand (TRAIL); (iv) upon contact with cancer cells, synNotch T cells prompt T cells to produce antibodies (e.g., αPD-1, αCTLA-4, or αCD3/CD19 BiTE) against specific immune checkpoints (ICs) or antigens, enhancing the efficacy of immunotherapy [7]. SynNotch T cells have shown good therapeutic effects against a variety of solid tumors.

In a series of subsequent studies, synNotch was combined with CAR-T cells to give the cells more specificity. In 2020, the Lim group deployed multiple synNotch in the same T cell to generate a complex combined sensing circuit [122]. Specifically, the authors designed a diverse library of multi-receptor cell recognition circuits by using synNotch to transcriptionally interconnect multiple molecular recognition events. These synthetic circuits allow engineered CAR-T cells to integrate extracellular and intracellular antigen recognition, and they achieve precise recognition by integrating up to three antigens with positive or negative logic, providing a powerful and precise recognition tool for CAR-T cells [122]. At the same time, various groups conducted therapeutic studies on solid tumors (e.g., glioma, mesothelioma, ovarian cancer), demonstrating that synNotch CAR-T cells produce a stronger anti-tumor effect and have greater specificity than conventional CAR-T cells. Conventional receptor tyrosine kinase-like orphan receptor 1 (ROR1)-targeted CAR-T cells not only lyse ROR1^+^ tumor cells but also attack ROR1^+^ normal stromal cells, which may cause therapeutic iatrogenic toxicity [123, 124]. Srivastava et al. developed ROR1-targeted CAR T cells expressiong synNotch  receptors for epithelial cell adhesion molecules (EpCAM) or B7-H3, which  are expressed on tumor cells but not on normal stromal cells [125]. In mouse and human solid tumor models, these synNotch CAR-T cells selectively killed EpCAM^+^ ROR1^+^ or B7-H3^+^ ROR1^+^  tumors cells  but not killed EpCAM^−^ROR1^+ ^cells or B7-H3^−^ROR1^+^ cells in normal tissues, resulting in tumor regression without toxicity [125]. Thus, this strategy safely targets tumors while sparing normal stromal cells, greatly reducing the extratumoral toxic effects of conventional CAR-T cell therapy [125]. In 2021, Choe et al. developed a synNotch CAR-T cell system whose synNotch receptor recognizes a specific priming antigen, such as the heterogeneous but tumor-specific glioblastoma neoantigen epidermal growth factor receptor splice variant III (EGFRvIII). After it is primed, the CAR-T cells are locally induced to express a second chimeric receptor targeting two more homogeneous tumor-specific antigens [EPH receptor A2 (EphA2) antigen or IL13Rα2 antigen] so as to switch on their highly specific killing program [126]. These synNotch CAR-T cells specifically recognized and killed EGFRvIII^+^EphA2^+^*/*IL13Rα2^+^glioblastoma cells while sparing healthy tissues [126].

Alkaline phosphatase placental-like 2 (ALPPL2), a tumor-specific antigen, is highly expressed in a spectrum of solid tumors (e.g., mesothelioma, ovarian cancer). Hyrenius-Wittsten et al. designed a synNotch CAR-T cell that targets ALPPL2 and another tumor-associated antigen [e.g., melanoma cell adhesion molecule (MCAM), mesothelin, or human epidermal growth factor receptor 2 (HER2)]. In mouse models of human mesothelioma and ovarian cancer, the synNotch CAR-T cells exerted superior control over tumor burden compared with traditional CAR-T cells, and they maintained long memory and a non-exhausted phenotype [127]. In neuroblastoma, Moghimi et al. engineered a specific synNotch protein on the surface of T cells to recognize the disialoganglioside (GD2) antigen [128]. When T cells recognized GD2, the synNotch protein instructed them to activate their CAR-T properties, allowing them to recognize a second antigen, B7-H3 [128]. These T cells followed these specific instructions to kill neuroblasts that carry both GD2 and B7-H3 [128].

In general, the above studies confirm that T cells, especially CAR-T cells, that contain engineered synNotch are better able to control solid tumors than conventional T cells. Thus, the synNotch system is a advantageous tumor recognition strategy that may navigate the concurrent challenges of specificity and heterogeneity to increase the therapeutic benefits of T cells against tumors, especially solid tumors (Fig. 3).

## Dysregulated Notch signaling in the tumor microenvironment and targeting it for cancer immunotherapy

The TME strongly affects responsiveness to immunotherapy, indicating that it plays a crucial role in accelerating or inhibiting cancer progression [129, 130]. Numerous studies have shown that tumor cells, stromal cells (e.g., cancer-associated fibroblasts, pericytes, mesenchymal stromal cells), as well as extracellular matrix (ECM) and secreted molecules in the TME (e.g., growth factors, cytokines, chemokines, and extracellular vesicles) can affect the infiltration and effector functions of immune cells, thus regulating tumor progression [131]. Compelling evidence indicates that alterations (activation or inhibition) in Notch signaling of tumor cells or stromal cells influence the effector functions of immune cells that infiltrate the TME, making Notch signaling a promising target in cancer immunotherapy.

### Dysregulated Notch signaling in tumor cells affects immune cell function in the TME

In BC patients, Jagged1 expression correlated with tumor progression. High Jagged1 expression correlated positively with infiltration of stromal M2-TAMs, which predicts poor patient survival and resistance to aromatase inhibitor therapy. However, BC cells pretreated with GSI and co-cultured with macrophages significantly inhibited the polarization of macrophages into M2-TAMs [132]. In BC, high expression of a long noncoding RNA, Linc00514, also promoted the expression of Jagged1, which in turn activated Notch signaling to promote the secretion of IL-4 and IL-6 from BC cells; these events then induced M2 polarization of macrophages. This suggests that activation of Notch signaling mediated by Jagged1 positively promotes M2-TAM polarization [133]. In multiple spontaneous BC models (e.g., 4T1 BC, PyMT-A BC), overexpression of tumor-derived Jagged1 promoted tumorigenesis [134]. By utilizing genetically engineered murine models of mammary-gland-specific Jagged1 overexpression or knockout mice, the researcher found that Notch activation by tumor-derived Jagged1 promoted the secretion of multiple cytokines (e.g., IL-6, WISP1) and TAM recruitment; the proliferation and tumoricidal activity of T cells were then inhibited, partially through upregulation of the T cells’ PD-1 [134]. Also, the combination of Notch inhibitor (GSI) with ICBs (αPD-1) significantly inhibited tumor growth in TNBC [134]. In pancreatic ductal adenocarcinoma (PDAC) patients, high Jagged1 expression in PDAC cells was associated positively with CD68^+^ macrophage infiltration and decreased patient survival [135]. Zhang et al. have shown that the upregulated expression of Dll1 in BC cells induces long-term normalization of tumor vascular and promotes the accumulation of CD8^+^ T cells and the polarization of M1-TAMs [136]. By recruiting 130 patients with invasive BC for bioinformatics and statistical analysis, the researcher found that high expression of Dll3 was associated with poor survival and with high levels of Treg cell infiltration [137]. High infiltration of tumor-associated neutrophils (TANs) was associated with immune tolerance and dismal prognosis in epithelial ovarian cancer (EOC) [138]. High expression of Jagged2 in tumor cells enhanced TAN infiltration, in turn inhibiting CD8^+^ T cell cytotoxicity. Blockade of Notch signaling [anti-Jagged2 antibody or LY3039478 (γ-secretase inhibitor)] reactivated CD8^+^ T cell-mediated anti-tumor properties, inhibiting tumor progression [138]. The above studies show that, in BC, PDAC, and EOC, high expression of Notch ligands (mainly Jagged1, Jagged2, and Dll3) of tumor cells promotes an immunosuppressive microenvironment in the TME, eventually allowing tumors to tolerate immunotherapy.

In addition to Notch ligands, abnormal expression of tumor-derived Notch receptors and downstream signaling genes affects the infiltration of immune cells and therefore tumor progression. By analyzing tumor samples from 152 patients with hormone receptor-positive and -negative phenotypes (luminal and triple-negative/basal-like) of BC, the author found that low mRNA levels of Notch receptors (mainly *Notch1*, *Notch2*, and *Notch4*) mainly in tumor cells were associated with higher infiltration of Treg cells into the tumors, predicting poor prognosis and poor survival [139]. However, in murine B16F10 melanoma models with subcutaneous and lung metastases, ectopic over-expression of Notch1 in B16F10 cells accelerated tumor progression and promoted tumor immunosuppression by upregulating TGF-β1. Specifically, forced high expression of Notch1 in B16F10 cells reduced the release of IFN-γ into the TME and inhibited the infiltration of CD8^+^ T cells and NK cells, while enhancing Treg cell and MDSC infiltration in vivo. PD-1 of CD4^+^ cells and CD8^+^ T cells were upregulated, accelerating T cell exhaustion [140]. In a mouse TNBC model, loss of function of ubiquitin-specific peptidase 9x-linked (USP9x) in tumor cells abolished NICD activation reduced the production of proinflammatory cytokines (e.g., CCL2, IL-1β), which further reduced the tumor inflammation through inhibiting the infiltration of CD206^+^ TAMs and Treg cells, augmenting the anti-tumor immune response through increase the infiltration of CD8^+^ T cells, suppressing BC tumor cell growth in vivo [141]. In a Tgfbr1/Pten knockout mouse model of HNSCC, Notch1 − Hes1 signaling was activated [19]. A γ-secretase inhibitor-DAPT, which inhibited Notch signaling, significantly decreased the burden of HNSCC tumors in that model [19]. Flow cytometry analysis demonstrated that the γ-secretase inhibitor also reduced the infiltration of MDSCs, TAMs, and Tregs into the spleen, draining lymph nodes and the TME as well as decreasing the expression of ICs (e.g., PD-1, CTLA-4, TIM-3, and LAG-3) in T cells in the circulation and tumor TME [19]. This study suggests that blocking Notch1 − Hes1 signaling in HNSCC might be an effective way to reduce immunosuppression and enhance therapeutic efficacy [19] (Table 2).

In the glioma TME, tumor cells escape immune surveillance and increase invasiveness by reducing Notch signaling. Specifically, loss of Notch signaling (mainly Notch1, Notch2, RBP-J, and Hey1) in glioma cells suppressed the expression of MHC-I and cytokines [e.g., C-X-C motif chemokine ligand 9 (CXCL9) and IL-15], reduced the recruitment of anti-tumor immune cells (e.g., CD8^+^ T cells), but favored the infiltration of microglia and pro-tumor TAMs [142]. In gastric cancer (GC) patients, both tumor tissue and peripheral blood showed significantly higher expression of Notch receptor (*NOTCH1*, *NOTCH2*) mRNA than normal human gastric tissue, and they also had higher proportions of Treg cells and Th17 cells [143]. Inhibiting Notch signaling with DAPT significantly suppressed Treg cell function in GC patients [143]. Another group also found that high Notch receptor (*NOTCH3*) expression was a poor prognostic factor when compared with 395 other genes in GC patients [144]. Specifically, high expression of Notch3 was associated with lower infiltration of anti-tumor immune cells (e.g., activated CD8^+^ T cells) and higher infiltration of immunosuppressive cells (e.g., Treg cells, M2-TAMs). In addition, high expression of Notch3 was accompanied by increased expression of a series of ICs [e.g., CD276, adenosine Aa2a receptor (ADORA2A)], resulting in a dampened anti-tumor immune response [144]. In addition to solid tumors, abnormal expression of Notch signaling in tumor cells of hematologic malignancies can also affect the infiltration of immune cells. For example, in diffuse large B cell lymphoma (DLBCL), mutation or knockdown of lysine methyltransferase 2D (KMT2D) in tumor cells indirectly activated Notch signaling (increased NICD protein), boosted the expression of downstream molecules (e.g., MYC and TGF-β1), and accelerated tumor progression by recruiting Treg cells [145]. Also in DLBCL, another group found that mutations in histone acetylation-related molecules [CREB binding protein (CREBBP) or E1A binding protein p300 (EP300)] in tumor cells contributed to tumor progression through indirectly upregulate Notch signaling [146]. Mechanistically, CREBBP or EP300 mutations indirectly activate Notch signaling (increased NICD protein, HEY1,and HEY2 mRNA) and downstream CCL2 − colony-stimulating factor 1(CSF1) in tumor cells, altering macrophage polarization into M2-TAMs and accelerating tumor progression[146] (Table 2). Based on these findings, we conclude that the abnormally expression of Notch receptors and their downstream signaling molecules in tumor cells affects tumor progression partially by regulating immune cells infiltration. Meanwhile, the specific regulatory mechanism of Notch signaling in tumor cell is complex and context-dependent (Table 3).

**Table 3:** Role of Notch signaling in regulating the tumor microenvironment

Regulators	Cancer type	Up/down-regulated	Cell type	Function	References
Jagged1	BC	Up	Tumor cell	Positively correlated with M2-TAMs infiltration	[132, 133]
Jagged1	TNBC	Up	Tumor cell	Positively correlated with TAMs infiltration; Negatively correlated with T cell cytotoxicity activity	[134]
Jagged1	PDAC	Up	Tumor cell	Positively correlated with CD68^+^ macrophages infiltration	[135]
Dll1	BC	Unknown	Tumor cell	Positively correlated with accumulation of CD8^+^T cells and the polarization of M1-TAMs	[136]
Dll3	BC	Up	Tumor cell	Positively correlated with Treg cell infiltration	[137]
Jagged2	EOC	Up	Tumor cell	Positively correlated with tumor-associated neutrophils; Negatively correlated with CD8^+^T cell infiltration	[138]
Notch1; Notch2; Notch4	BC	Down	Tumor cell	Negatively correlated with Treg cells infiltration	[139]
Notch1	Melanoma	Unknown	Tumor cell	Negatively correlated with CD8^+^ T cells and NK cells infiltration; Positively correlated with MDSCs and Treg cells infiltration	[140]
Notch1	TNBC	Unknown	Tumor cell	Positively correlated with CD206^+^ TAMs and Treg cells infiltration; Negatively correlated with the infiltration of CD8^+^ T cells	[141]
Notch1	HNSCC	Up	Tumor cell	Negatively correlated with MDSCs, TAMs, Treg cells infiltration and the expression of immune checkpoint molecules (e.g., PD-1, CTLA-4, TIM-3, and LAG-3)	[19]
Notch1; Notch2; RBP-J; Hey1	Glioma	Down	Tumor cell	Positively correlated with the recruitment of anti-tumor immune cell populations, such as CD8^+^T cells; Negatively correlated with the recruitment of microglia and TAMs	[142]
Notch1; Notch2	GC	Up	Tumor cell	Positively correlated with Treg cells and Th17 cells infiltration	[143]
Notch3	GC	Up	Tumor cell	Positively correlated with Treg cell and M2-TAM infiltration and the expression of immune checkpoints (CD276, ADORA2A); Negatively correlated with activated CD8^+^ T cell infiltration	[144]
Notch	DLBCL	Up	Tumor cell	Positively correlated with M2-TAMs polarization and infiltration	[146]
Notch1	CRC	Up	Epithelial cell	Positively correlated with recruitment of TGF-β-dependent neutrophils	[147]

BC: Breast cancer; TNBC: Triple-negative breast cancer; PDAC: Pancreatic ductal adenocarcinoma; EOC: Epithelial ovarian cancer; HNSCC: Head and neck squamous cell carcinoma; GC: Gastric cancer; DLBCL: Diffuse large B cell lymphoma; CRC: Colorectal cancer

### Dysregulated Notch signaling in stromal cells affects immune cell function in the TME

As well as tumor cells, stromal cells in the TME can also regulate immune cell infiltration and function through Notch signaling. In the TME of KRAS^G12D^-driven CRC, Jackstadt et al. found that epithelial Notch1 signaling was critical in disease subtypes with the poorest prognoses and liver metastasis of CRC [147]. Mechanistically, activation of Notch1 in epithelial cells promoted the secretion of TGF-β into the TME, increased the recruitment of TGF-β-dependent neutrophils, and inhibited the anti-tumor function of CD8^+^ T cells [147]. In contrast, recruitment of neutrophils was significantly inhibited by 1D11 (a ligand-trapping antibody targeting TGF-β1/2/3) and then suppressed CRC tumor liver metastasis [147] (Table 2). As the author demonstrated that epithelial Notch1 signaling was critical for the secretion of TGF-β [147], we speculate that blocking Notch1 signaling of intestinal epithelial cells might be a potential strategy for inhibiting CRC metastasis. It could therefore suggest a clinical treatment for liver metastasis in CRC.

## Potential role of Notch signaling in tumor immunity mediated by gut microbiota

Gut microbiota (GM) (e.g., bacteriophages, viruses, bacteria, helminths, and fungi) are microorganisms in the gastrointestinal tract of humans or mammals, with bacteria accounting for more than 99% of the species [148]. Gut microbiota can directly or indirectly regulate immune cells to affect tumor progression [149–151]. For example, gut microbes or their metabolites can modulate the responses of immune cells (e.g., ILC3s, Th1 cells, and CD8^+^ T cells) to control CRC progression [152–154]. In an MHCII^ΔILC3^ murine CRC model, it was demonstrated that MHC II^+^ ILC3s supported the colonization of gut microbiota that boosted the anti-tumor properties of Th1 and T-bet^+^ CD8^+^ T cells [152]. The colonized microbes also regulated the differentiation and activation of Treg cells, Th1 cells, and Th17 cells to control intestinal disease (e.g., cancer, autoimmune diseases) [153, 155–157]. Metabolites of gut microbiota (e.g., butyric acid, pentanoate, and butyrate) also induced Treg cell differentiation [158], increased the secretion of anti-tumor cytokines (e.g., IFN-γ and TNF-α) by CD8^+^ T cells, and enhanced the anti-tumor responses of antigen-specific CTLs and CAR-T cells [159]. In collaboration with the Wang lab, we found that feeding black raspberries, a natural product, significantly induced distinct changes in murine gut microbiota, increased the abundance of anti-inflammatory microbial species (e.g., *Akkermansia* and *Desulfovibrio*), activated anti-tumor immune cells (e.g., NK cells), and enhanced those cells’ anti-tumor immune responses [160–163]. Conversely, dysbiosis of gut microbiota in mice increased susceptibility to colon tumors because it overstimulated CD8^+^ T cells, which in turn promoted chronic inflammation and early T cell exhaustion, thereby reducing the cells’ anti-tumor immune response [154].

Gut microbiota have profound effects on host physiology through classical signaling (e.g., Notch signaling, WNT signaling, and PI3K − Akt signaling) [164]. In recent years, interactions between host microbiota and Notch signaling have also been revealed. Roy et al. [165] found that controlling the hyperactivation of Notch signaling was important for preventing intestinal inflammation mediated by C*itrobacter rodentium* in humans and mice. The activity of microbiota, determined by innate immune signaling, correlated with activation of Notch signaling in the intestinal epithelium, suggesting that Notch signaling played a role in maintaining gut homeostasis and that its dysregulation would lead to chronic inflammation or cancer [166]. Inhibition of Notch1 activation by indoleamine 2,3-dioxygenase-1 (IDO1) in mice significantly increased both the thickness of the intestinal mucus layer and the proportion of intestinal *Akkermansia muciniphila* and *Mucispirillum schaedleri*. Additionally, mice that received IDO1 to inhibit Notch1 activation had 85% fewer ileal bacteria after a challenge with enteropathogenic *E. coli* compared with control mice [167]. In other studies, NKp46^+^ ILC3s played a positive role in controlling tumor progression [168, 169], and Notch signaling activation proved crucial for sustaining AhR-mediated production of NKp46^+^ ILC3s in the lamina propria [75]. From the above study, we speculate that Notch signaling plays a key role in mediating the anti-tumor effects of gut microbiota on NKp46^+^ ILC3s, but the specific regulatory mechanism requires further in-depth study. In general, we speculate that Notch signaling could have a regulatory role in tumor immunity mediated by gut microbiota, such as by boosting NKp46^+^ ILC3 numbers. Therefore, a deeper understanding of the potential functional interactions of Notch signaling-mediated immune cells with gut microbes may provide new strategies for developing innovative immunotherapies against cancer.

## Strategies for targeting Notch signaling in cancer immunotherapy

In the above discussion, we concluded that Notch signaling, including ligands (e.g., Jagged1, Dll1, and Dll4), receptors (e.g., Notch1, Notch2), and downstream utility molecules (e.g., RBP-J, Hes1), is directly involved in the regulation of immune cells’ anti- or pro-tumor immune responses in various ways. At the same time, abnormal expression of Notch signaling in tumor cells or stromal cells can regulate immune cell infiltration, resulting in an immunosuppressive TME and accelerated tumor progression.

Targeting Notch signaling for cancer immunotherapy could be achieved by: (1) combining oncolytic virotherapy with inhibition of Notch signaling to efficiently inhibit the proliferation and other properties of immunosuppressive cells (e.g., MDSCs); (2) customizing the delivery of Notch activators into TAMs via nanoparticles to promote the M1 polarization of TAMs and activate CD8^+^ T cells and ultimately remodel the TME; (3) combining Notch drugs with ICBs to synergistically enhance anti-tumor immunotherapy; (4) customizing the synNotch circuit into CAR cells (e.g., CAR-T cells, CAR-NK cells, or CAR-Macrophages) to enhance the precision of CAR immune cell therapy. Below, we discuss these potential strategies in more detail.

### Oncolytic virotherapy combined with inhibition of Notch signaling

Oncolytic virotherapy, an emerging cancer immunotherapy, has received extensive attention in recent years [170]. One of the most widely investigated oncolytic viruses is oHSV, as it efficiently lyses tumor cells while leaving normal cells unscathed. In 2015, the US Food and Drug Administration (FDA) approved the first oncolytic HSV—oHSV-talimogene laherparepvec (T-VEC)—for treating melanoma patients (Clinical Trial.gov identifier: NCT02173171) [171]. Now, oHSV is used to treat glioblastoma (GBM) [93], melanoma [171], breast cancer [172], and ovarian cancer [92]. One study from our group showed that customized oHSV had significant efficacy against GBM in pre-clinical mouse models [173]. Our customized OV-CDH1 oncolytic virus was able to spread into tumors and lyse tumor cells more effectively than control oHSV. It also selectively prevented KLRG1^+^ NK cells from lysing OV-CDH1-infected tumor cells, improving the efficacy of cancer virotherapy [173]. In two recent studies, we customized an oHSV to express a full-length anti-CD47-IgG1 antibody [92, 93]. After that OV-αCD47 infected murine GBM or ovarian tumor in vivo, it lysed tumor cells, released αCD47 into the TME, and induced antibody-dependent cellular cytotoxicity (ADCC) of NK cells and antibody-dependent cellular phagocytosis (ADCP) of macrophages, thus cooperatively enhancing the therapeutic efficacy of cancer virotherapy [93].

**Fig. 4:**
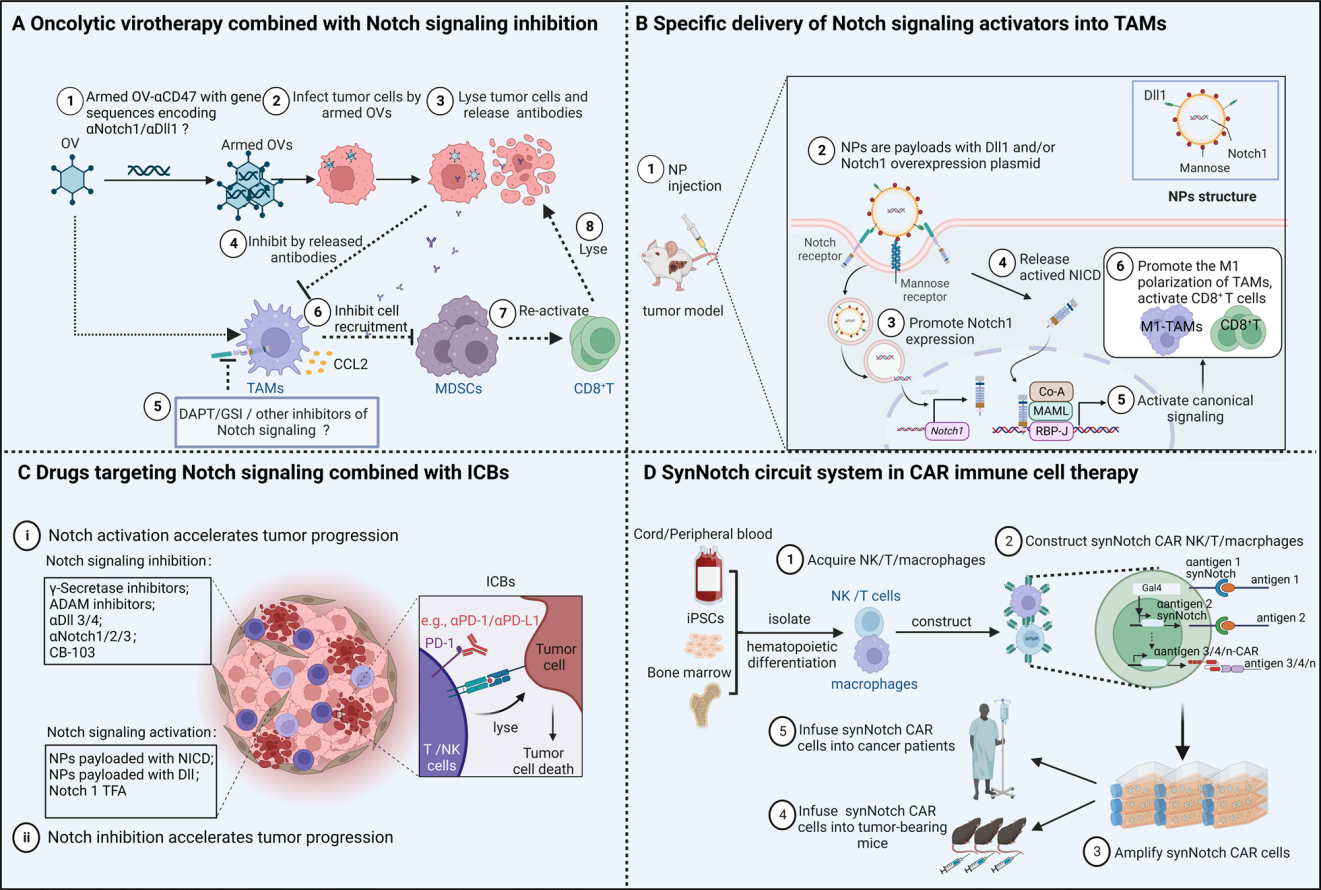
Strategies for targeting Notch signaling in cancer immunotherapy. **A:** Oncolytic virotherapy combined with Notch signaling inhibition. αCD47 can be incorporated into OVs along with gene sequences of antibodies that inhibition of Notch signaling (e.g., αNotch1, αDll1). Also, combination of DAPT, GSI, or other inhibitors of Notch signaling with OVs inhibits the CCL2 secretion of TAMs and the recruitment of MDSCs, and reactivation of CD8^+^ T cells; **B:** Specific delivery of Notch signaling activators into TAMs. Dll1 ligand and Notch1 overexpression plasmid are packaged into mannose-NPs to specifically target TAMs,  promoting the polarization of TAMs to M1-TAMs and activating CD8^+^ T cells; **C:** ICBs combined with drugs targeting Notch signaling. **D:** The synNotch circuit system can be incorporated into CAR immune cells for immunotherapy. Several types of CAR cells can be used to increase anti-tumor capacity. They include synNotch CAR-T cells, synNotch CAR-NK cells, or synNotch CAR-M cells that recognize multiple antigens. These synNotch strategies can enhance the specificity of CAR cells and improve their anti-tumor functions. Figure was created with BioRender.com

In the TME of GBM, oHSV infection abnormally activates Notch signaling, causing TAMs to secrete large amounts of cytokines (e.g., CCL2, IL-10). It then recruits MDSCs to inhibit the therapeutic effect of oncolytic virotherapy [98]. Adding GSI, a pharmacological blocker of Notch signaling, rescued the oHSV-induced immunosuppressive TME and activated CD8^+^ T cell-dependent anti-tumor memory responses, resulting in therapeutic benefits [98]. Therefore, by combining our previously developed oncolytic viruses (e.g., OV-αCD47) with gene sequences encoding antibodies (e.g., αNotch1, αDll1) that block Notch signaling or by combining DAPT, GSI, or other inhibitors of Notch signaling with OVs, we can inhibit cytokine (e.g., CCL2) secretion of TAMs and the recruitment of MDSCs, reactivating CD8^+^ T cells for cancer immunotherapy. These combination therapies have important implications for the clinical treatment of solid tumors (e.g., glioma) that respond positively to inhibition of Notch signaling (Fig. 4A).

### Encapsulating drugs that target Notch signaling into nanoparticles and specifically delivering them to TAMs in the TME

Targeted delivery of drugs into specific immune cells or the TME to transform “cold tumor” into “hot tumor” is an emerging and promising strategy for cancer immunotherapy [174]. In recent years, nanoparticles (NPs) have shown great clinical potential in drug delivery systems, as they can accurately and effectively deliver many types of drugs (e.g., oligonucleotides, siRNAs, or protein-based drugs) into TAMs of the TME. For example, siRNAs that modulate NF-κB signaling [175] and VEGF signaling [85] can be payloaded into polymeric NPs; anti-CSF-1R siRNA can be incorporated into lipid-based NPs [176]; and cytosine-phosphate-guanine (CpG) [Toll-like receptor 9 (TLR9) agonist] can be payloaded into carbon NPs [177]. These NPs have been successfully delivered into TAMs of the TME, affecting the cells’ functionality.

Our group and others have found that activating Notch signaling in TAMs of the TME (e.g., in murine lung cancer) promotes TAM polarization into proinflammatory M1-TAMs, thereby increasing the infiltration of CD8^+^ T cells, further inhibiting tumor progression [88, 91]. By integrating Notch-activating ligands (e.g., Dll1) and/or Notch1 overexpression plasmid into mannose-NPs, Notch signaling can be activated in TAMs but not in other cells. Thus, TAMs can be polarized into M1-TAMs, fulfilling the goal of remodeling the TME to improve tumor immunotherapy (Fig. 4B).

### Combining ICBs with drugs that target Notch signaling

ICs are key regulators of immune system suppression [178]. ICBs (e.g., αCTLA-4, αPD-1/αPDL-1) can block inhibitory checkpoints, thereby unleashing suppressed anti-tumor immune responses [179]. In recent years, ICB-based immunotherapy, including αPD-1/αPD-L1 and αCTLA-4, has significantly improved the survival rates of patients with metastatic solid tumors, especially melanoma and lung cancer [180, 181]. Also, ICBs have correlated significantly with Notch signaling changes (activation or inhibition) in various tumors. Activation of Notch signaling in human neuroendocrine (NE) SCLC cell lines induced low NE differentiation and increased intrinsic tumor immunity [182]. Activation of Notch signaling was found to be an important predictor of the clinical benefit of ICB used in two relapsed SCLC cohorts [182]. In CRC, Notch signaling mutations in tumor cells were associated with the enrichment of cytotoxicity-related molecules (e.g., GZMB and PRF1) but also exhaustion-related molecules (e.g., PD-1) [183]. We found that, in some tumors (e.g., TNBC), ICBs combined with GSI inhibited tumor progression [134]. In other tumors (e.g., HNSCC, GC), high Notch expression promoted the expression of ICs, indicating that ICBs may have better therapeutic effects in these cancer patients with high Notch expression compared to those with low expression [19, 140, 146].

In summary, activation or inhibition of Notch signaling in tumor cells can affect the expression of ICs, thus likely modulating the therapeutic effect of ICBs. However, a previous study found that, in anti-tumor T cells, activation of Notch signaling enhanced the cells’ cytotoxicity but could also promote the expression of PD-1, potentially promoting T cell exhaustion. Therefore, we should adopt different synergistic therapeutic strategies in different contexts: (i) For a TME in which inhibition of Notch signaling enhances IC expression (e.g., PD-1), we could combine clinically used inhibitors that target Notch signaling (e.g., γ-secretase inhibitors, ADAM inhibitors) with ICBs (e.g., αPD-1/αPDL-1) to obtain synergistic anti-tumor effects; (ii) For a TME in which activation of Notch signaling enhances the expression of ICs (e.g., PD-1), we could develop NPs loaded with a Notch signaling activator [e.g., NICD, Dll1, Dll3, Dll4, or Notch homolog 1-translocation-associated (Notch1 TFA)] specifically into target cells (e.g., T cells). Combining these NPs with ICBs (e.g., αPD-1/αPDL-1) would produce additive or synergistic anti-tumor activity (Fig. 4C).

### Developing a synNotch circuit for CAR immune cell therapy

CAR (chimeric antigen receptor) protein is a synthetic cell surface receptor that confers immune cells (e.g., T cells, NK cells, and macrophages) with specific anti-tumor properties that can target corresponding antigenic proteins [184]. CAR-T cells have achieved unprecedented success with some hematological malignancies, and a couple of products have already been approved by the US FDA [185, 186]. Other CAR immune cells [including CAR-NK cells, CAR-NKT, CAR-macrophage (CAR-M), and CAR-γδT] have been approved for or are in clinical trials, as documented in our recent review of CAR immune cells’ great potential for improving cancer immunotherapy [184]. Because allogenic CAR-NK cells are efficacious against tumor cells but do not produce cytokine storms or graft-versus-host disease (GVHD) [187], they are being developed into ‘off the-shelf’ drugs for immunotherapy. Using animal models, we obtained a significant anti-tumor effect when we recently used ‘off-the-shelf’ human EGFR-CAR-NK cells and human PSCA-CAR-s15NK cells to treat solid tumors (e.g., glioma and pancreatic cancer) [188, 189]. Also, CAR-M cells have attracted great interest as potential immunotherapies in recent years. Researchers have found that modifying human macrophages with specific CARs can improve the presentation of tumor antigens (especially those on solid tumors) and increase macrophages’ phagocytic activity. These CAR immune cell-mediated tumor therapies have produced good results or shown great potential in the majority of hematological tumors and some non-homogeneous solid tumors. However, very few antigens are truly tumor-specific, and thus, conventional CAR-T cell therapies often cause lethal toxicities such as on-target, off-tumor cross-reaction of CAR cells with normal tissues; they also have poor specificity [126, 190–192]. In fact, the majority of tumor antigens are often expressed heterogeneously, and treatment with conventional CAR cells allows antigen-negative tumor cells to escape immune surveillance [193]. Therefore, there is an urgent need to develop a new tumor recognition system of CAR cells—one that can recognize tumor cells carrying multiple antigens—to deal with tumor heterogeneity and thereby increase the therapeutic effectiveness of CAR immune cells against solid cancers.

Recently, the synNotch system, developed by the Lim group at the new frontier of cancer research, was launched. This system can accurately teach T cells (especially CAR-T cells) to recognize two or three antigens of solid tumors (e.g., mesothelioma, ovarian tumor, or glioblastoma) [122]. In the section—“SynNotch can be used as a tool to increase T cell cytotoxicity and specificity”, we explained how the synNotch system enhances anti-tumor specificity mediated by T cells (mainly CAR-T cells), especially in solid tumors. SynNotch circuits allow CAR-T cells to integrate extracellular and intracellular antigen recognition signals and accurately identify and kill tumor cells, as they use positive or negative logic to combine two or multiple different antigens [122, 126–128]. Important future developments will likely include: (i) synNotch circuits that can simultaneously recognize multiple different antigens on tumor cells by CAR-T cells to precisely kill highly heterogeneous solid tumors; (ii) synNotch circuits that can simultaneously recognize multiple antigens on tumor cells by CAR-NK or CAR-M cells; (iii) CAR immune cells can persist in vivo and kill solid tumors by more accurately dissolving or swallowing tumor cells (Fig. 4D).

## Conclusions and perspectives

In this review, we summarized recent advances in understanding the mechanism of Notch signaling in immune cells and its roles in immune responses. It is important to acknowledge, however, that this area of investigation is complex, and much is still to be learned. More comprehensive understanding of the biological function of Notch signaling in immune responses should facilitate the development of Notch targets for more precise tumor immunotherapy.

We also proposed rational strategies for ameliorating cancer immunotherapy based on targeting Notch signaling, including the development of: (i) Notch inhibitors packaged into oncolytic viruses and released into the TME, where they effectively inhibit the recruitment of immunosuppressive cells such as MDSCs; (ii) targeted delivery of Notch activators into TAMs via NPs to re-educate TAMs and reprogram them to the M1 phenotype to ultimately remodel the TME; (iii) combinations of Notch drugs with ICBs to synergistically enhance the effects of anti-tumor immunotherapy, as ICB therapy has been a breakthrough in cancer treatment. As noted above, Notch signaling not only alters IC expression patterns in multiple cancers but also modulates ICB efficacy in some preclinical animal models. At present, drugs targeting Notch signaling are in clinical trials for several solid tumors, but there are few studies on combining ICBs with drugs that affect Notch signaling. Based on previous investigations, we speculate that such combinations could be tested in clinical trials for their synergistic anti-tumor effects in humans; (iv) transformation of CAR immune cells with the synNotch circuit to enhance synergistic therapeutic effects and CAR cell safety. 

However, the therapeutic interventions of Notch signaling are challenging because the undesired “on-target, off-tumor” activity may potentially lead to significant toxicity. Also, non-specific intervention of Notch signaling can have the opposite effect on controlling tumor development because of targeting both tumor cells and immune cells. For example, the intervention to inhibit tumor cells may also suppress immune responses to tumor cells. Thus, a deeper understanding of Notch signaling in different cell types and the interactions between the Notch signaling pathway and other pathways may contribute to the development of more innovative and precise targeted therapeutics that will provide better clinical outcomes in cancer patients.

## Data Availability

This is a review article that does not have original data.

## Abbreviations

TME: Tumor microenvironment
NPs: Nanoparticles
TAMs: Tumor-associated macrophages
ICBs: Immune checkpoint blockers
CAR: Chimeric antigen receptor
MDSCs: Myeloid-derived suppressor cells
DCs: Dendritic cells
synNotch: Synthetic Notch
PD-1: Programmed cell death protein 1
CTLA-4: Cytotoxic T lymphocyte-associated antigen-4
IL-2: Interleukin 2
IL-12: Interleukin 12
ER: Endoplasmic reticulum
NECD: Extracellular domain of Notch receptors
NICD: Intracellular domain of Notch receptors
RBP-J: Recombination signal binding protein for immunoglobulin kappa (к) J region
MAML: Mastermind-like
pre-Notch receptors: precursors of Notch receptors
SERCA: Sarcoendoplasmic reticulum Ca^2+^-ATPase
CRC: Colorectal cancer
PCa: Prostate cancer
ESCC: Esophageal squamous cell carcinoma
mAb: Monoclonal antibody
ACC: Adenoid cystic carcinoma
PR: Partial response
SCLC: Small cell lung cancer
OS: Overall survival
PFS: Progression-free survival
ORR: Objective response rate
NSCLC: Non-small cell lung cancer
ADAM: A disintegrin and metalloprotease
TM: Transmembrane
HCC: Hepatocellular carcinoma
GSIs: γ-Secretase inhibitors
BC: Breast cancer
TNBC: Triple-negative breast cancer
HNSCC: Head and neck squamous cell carcinoma
EAC: Esophageal adenocarcinoma
NK cells: Natural killer cells
UCB: Umbilical cord blood
IL-7: Interleukin 7
Flt3: Fms-like tyrosine kinase 3
IL-15: Interleukin 15
GZMB: Granzyme B
IFN-γ: Interferon-gamma
KIRs: Killer Ig-like receptors
ILCs: Innate lymphoid cells
Tbx21: T-box transcription factor 21
ILC1s: Group 1 ILCs
ILC2s: Group 2 ILCs
ILC3s: RORγt-expressing ILCs
LTi cells: Lymphoid tissue-inducer cells
BM: Bone marrow
CLPs: Common lymphoid progenitors
AhR: Aryl hydrocarbon receptor
SI: Small intestine
HPC-1: Hematopoietic progenitor cell subpopulation one
M1-TAMs: M1-like tumor-associated macrophages
M2-TAMs: M2-like tumor-associated macrophages
Socs3: Suppressor of cytokine 3
SIRPα: Signal regulatory protein α
oHSV: Type 1 herpes simplex virus-based oncolytic virus
αCD47: Anti-CD47 antibody
kclTAMs: Kupffer cell-like TAMs
moTAMs: Monocyte-derived TAMs
IL-10: Interleukin 10
MDSCs: Myeloid-derived suppressor cells
PMN-MDSCs: Polymorphonuclear-MDSCs
M-MDSCs: Mononuclear MDSCs
STAT3: Signal transducer and activator of transcription 3
GSEA: Cybersort and Gene Set Enrichment Analysis
MCT2: Lactate import 2
APCs: Antigen-presenting cells
CCR7: CC-chemokine receptor 7
cDCs: Conventional DCs
LBP: Lycium barbarum polysaccharide
CTLs: Cytotoxic T lymphocytes
TCIM: Transcriptional and immune response regulator
EZH2: Enhancer of zeste homolog 2
TNF-α: Tumor necrosis factor alpha-like
IL-1β: Interleukin-1 beta
CLL: Chronic lymphocytic leukemia
ALL: Acute lymphoblastic leukemia
TRAIL: TNF-related apoptosis-inducing ligand
ICs: Immune checkpoints
ROR1: Receptor tyrosine kinase-like orphan receptor 1
EpCAM: Epithelial cell adhesion molecule
EGFRvIII: Epidermal growth factor receptor splice variant III
EphA2: EPH receptor A2
ALPPL2: Alkaline phosphatase placental-like 2
MCAM: Melanoma cell adhesion molecule
HER2: Epidermal growth factor receptor 2
GD2: Disialoganglioside
ECM: Extracellular matrix
PDAC: Pancreatic ductal adenocarcinoma
TANs: Tumor-associated neutrophils
EOC: Epithelial ovarian cancer
USP9x: Ubiquitin-specific peptidase 9x-linked
IL-1β: Interleukin-1 beta
CXCL9: C-X-C motif chemokine ligand 9
GC: Gastric cancer
ADORA2A: Adenosine A2a receptor
DLBCL: Diffuse large B cell lymphoma
KMT2D: Lysine methyltransferase 2D
CREBBP: CREB binding protein
EP300: E1A binding protein p300
CSF1: Colony-stimulating factor 1
GM: Gut microbiota
IDO1: Indoleamine 2,3-dioxygenase-1
FDA: Food and drug administration
T-VEC: OHSV-talimogene laherparepvec
GBM: Glioblastoma
ADCC: Antibody-dependent cellular cytotoxicity
ADCP: Antibody-dependent cellular phagocytosis
CpG: Cytosine-phosphate-guanine
TLR9: Toll-like receptor 9
NE: Neuroendocrine
CAR-M: CAR-macrophages
GVHD: Graft-versus-host disease
